# Associations of metabolic dysfunction-associated steatotic liver disease and significant fibrosis phenotypes with glomerular hyperfiltration defined by indexed eGFR

**DOI:** 10.3389/fnut.2026.1944355

**Published:** 2026-09-30

**Authors:** Wen Tian, Jiameng Wang, Xueling Xu, Qiang Li, Ya Zheng, Hong Lu

**Affiliations:** 1 The First Clinical Medical College, Lanzhou University, Lanzhou, China; 2 Department of Gastroenterology, The First Hospital of Lanzhou University, Lanzhou, China; 3 Gansu Province Clinical Research Center for Digestive Diseases, The First Hospital of Lanzhou University, Lanzhou, China

**Keywords:** body surface area, estimated glomerular filtration rate, glomerular hyperfiltration, liver fibrosis, metabolic dysfunction-associated steatotic liver disease

## Abstract

**Background:**

To examine associations of metabolic dysfunction-associated steatotic liver disease (MASLD) and significant fibrosis with glomerular hyperfiltration (GHF) defined from indexed estimated glomerular filtration rate (eGFR), accounting for body size.

**Methods:**

We analyzed 9,140 NHANES participants and 721 adults from a Chinese clinical study. Liver phenotypes were G1 (no steatosis/no significant fibrosis), G2 (MASLD without significant fibrosis), and G3 (MASLD with significant fibrosis). Primary GHF was indexed eGFR above the survey-weighted age- and sex-specific 95th percentile of a low-risk NHANES reference, applied unchanged to both studies. BMI-adjusted Model 3 was central; metabolic adjustment and alternative GHF definitions were sensitivity analyses.

**Results:**

In NHANES Model 3, G2 and G3 had higher odds of GHF than G1 (OR 2.19, 95% CI 1.57–3.07; OR 2.46, 1.51–4.00), whereas G3 did not differ from G2 (OR 1.12, 0.74–1.70). In the identical fasting subsample, further metabolic adjustment attenuated G2 and G3 versus G1 (OR 1.94 and 2.08), while G3 versus G2 remained near the null. In China, G2 versus G1 was null, whereas G3 had higher odds than G1 and G2; however, G3 included only 29 participants and 8 events. NHANES findings were generally robust to indexed P90, age/sex/BSA-residual, and de-indexed sensitivity definitions.

**Conclusion:**

In NHANES, MASLD phenotypes were associated with higher odds of GHF after accounting for body size, whereas significant fibrosis showed no clear incremental association beyond MASLD. The fibrosis-related association observed in the Chinese clinical study should be interpreted cautiously because of the small G3 subgroup and cross-population differences in GHF calibration. Prospective studies using measured GFR are needed to clarify the temporal and prognostic significance of GHF in MASLD.

## 1 Introduction

Metabolic dysfunction-associated steatotic liver disease (MASLD) is the nomenclature and diagnostic framework established by a multisociety consensus in 2023 for steatotic liver disease characterized by hepatic steatosis together with at least one cardiometabolic risk factor (1). Current European and United States guidance emphasizes risk stratification and management across the cardiometabolic spectrum (2, 3). The underlying steatotic liver disease burden is high and continues to increase worldwide (4, 5), and non-alcoholic fatty liver disease (NAFLD)/MASLD is increasingly recognized as a multisystem disorder with important extrahepatic consequences (6–8). Longitudinal studies and meta-analyses have consistently linked NAFLD/MASLD with incident chronic kidney disease (CKD), although most available evidence has focused on relatively late renal outcomes such as eGFR decline, proteinuria, or CKD (9–14).

Glomerular hyperfiltration (GHF) generally refers to a filtration rate above physiological requirements and has been linked to altered intraglomerular hemodynamics, elevated single-nephron filtration, and early metabolic kidney stress (15–17). However, no uniform threshold for GHF has been established, and published studies differ substantially in how they account for age, sex, body size, and the method used to measure or estimate glomerular filtration rate (GFR) (18). Hyperfiltration has been associated with renal disease progression in diabetes (19), while human single-nephron studies demonstrate that higher single-nephron GFR can accompany obesity and structural kidney changes even in otherwise healthy adults (20). A systematic review further showed marked heterogeneity in GHF definitions and supported the methodological value of age- and sex-matched reference ranges (18).

Evidence linking MASLD to GHF remains limited. Abbate et al. reported an association between NAFLD and GHF among adults with metabolic syndrome (21), whereas Dalbeni et al. (22) found that liver fibrosis assessed by vibration-controlled transient elastography (VCTE) was associated with GHF in adults with MASLD. Conversely, a large Korean longitudinal cohort showed that baseline age- and sex-specific GHF was associated with subsequent development of NAFLD and progression of liver fibrosis, suggesting a potentially bidirectional or shared-metabolic relationship (23). Broader kidney-outcome studies have also related NASH or liver-fibrosis severity to lower kidney function, CKD, or albuminuria (24–26). Differences in GHF definitions, liver assessment methods, and study populations limit direct comparisons across these studies.

Body size is a particularly important methodological consideration when interpreting this association. Conventional eGFR is indexed to a body surface area (BSA) of 1.73 m^2^; when eGFR is de-indexed to mL/min, the value is multiplied by BSA/1.73 and may therefore increase mechanically in individuals with obesity or larger body size (27, 28). Obesity itself is also associated with higher measured GFR and renal plasma flow, consistent with a biological hyperfiltration phenotype (29, 30). In addition, serum creatinine is influenced by muscle mass, and creatinine-based eGFR remains subject to estimation error at high filtration levels and at extremes of body size (27, 31, 32). Contemporary CKD guidance likewise emphasizes appropriate interpretation of eGFR and filtration markers in clinical context (33). Accordingly, studies of MASLD and GHF should distinguish, as far as possible, body-size-related mathematical or measurement effects from genuine renal hemodynamic alterations.

Against this background, our primary objective was to examine the association between MASLD phenotypes and GHF defined using indexed eGFR, while minimizing potential bias related to body size. We prespecified two main hypotheses: first, participants with MASLD without significant fibrosis (G2) would have greater odds of GHF than participants without MASLD (G1); and second, participants with MASLD and concomitant significant fibrosis (G3) would have greater odds of GHF than those in G1. We further explored whether concomitant significant fibrosis was associated with additional odds of GHF by comparing G3 with G2.

## 2 Materials and methods

### 2.1 Study design and populations

This study comprised two complementary cross-sectional analyses and was reported in accordance with the Strengthening the Reporting of Observational Studies in Epidemiology (STROBE) statement (34). The population-based analysis used data from the US National Health and Nutrition Examination Survey (NHANES), combining the 2017-March 2020 pre-pandemic release with the August 2021–August 2023 release. The second analysis was a retrospective study of inpatient clinical records from the First Hospital of Lanzhou University.

The NHANES analysis included adults aged 20–79 years. This common adult age window was selected for the revised analysis to support stable, directly comparable 10-year age/sex reference strata (20–29 through 70–79 years) in both cohorts. GHF thresholds are strongly age dependent, and age- and sex-matched reference distributions are methodologically preferred (18, 35, 36). The upper limit of 79 years was an analytic choice rather than a clinical boundary; the 70-79-year low-risk NHANES reference strata were already the sparsest, so creating an additional ≥80-year empirical P95 stratum would have further reduced threshold stability. Participants were required to have completed the Mobile Examination Center assessment, not be pregnant, have valid high-quality liver transient elastography measurements, and have serum creatinine available for calculation of indexed eGFR. In the fully auditable revised flow, 27,493 merged records were reduced to 23,160 MEC-examined participants, 13,690 aged 20–79 years, 13,562 nonpregnant participants, 11,395 with known alcohol exposure below the MASLD threshold, 10,215 with valid CAP/LSM, and 9,461 with valid indexed eGFR; exclusion of 321 non-primary phenotype combinations yielded 9,140 participants in the primary G1/G2/G3 analysis (Figure 1).

**Figure 1:**
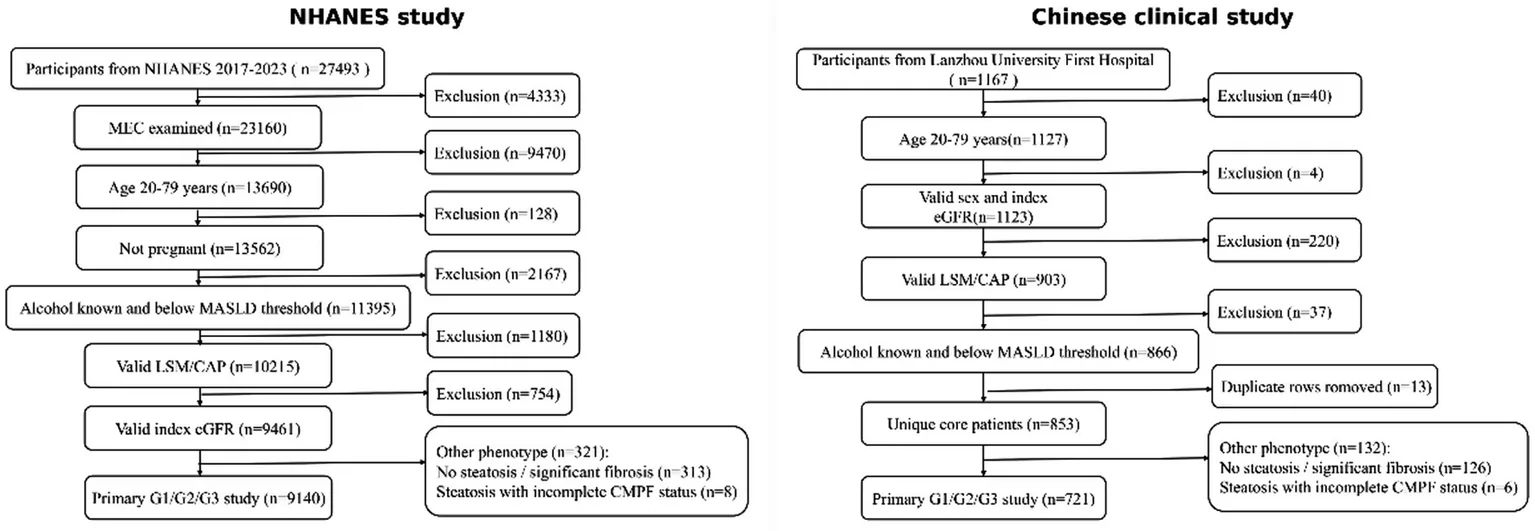
Participant selection and derivation of the primary analytic cohorts. The flow diagram reports exclusions at each step and explicitly identifies phenotype combinations outside the primary three-level analysis. Alcohol eligibility required known intake below the sex-specific MASLD threshold. G1, no steatosis/no significant fibrosis; G2, MASLD without significant fibrosis; G3, MASLD with significant fibrosis.

The Chinese clinical study included hospitalized adults evaluated at the First Hospital of Lanzhou University between July 2021 and April 2025 who underwent VCTE during routine clinical care. The cohort was not based on consecutive enrollment; the decision to perform VCTE was made by the treating physician according to each patient’s clinical condition. The same 20-79-year age window was applied for cross-study comparability. VCTE, serum creatinine, blood pressure, fasting glucose, lipids, and other metabolic variables were anchored to the first available measurement after admission. Serum creatinine was measured by the hospital clinical laboratory as part of routine inpatient testing. The revised flow began with 1,164 records: 1,127 were aged 20–79 years, 1,123 had valid indexed eGFR, 903 had valid CAP/LSM, 866 had known alcohol exposure below the MASLD threshold, 853 unique core patients remained after deduplication, and 721 belonged to G1/G2/G3 (Figure 1). The Chinese study was approved by the Ethics Committee of the First Hospital of Lanzhou University (LDYYLL2026-682) and conducted in accordance with the Declaration of Helsinki; NHANES protocols were approved by the National Center for Health Statistics Research Ethics Review Board, and all participants provided informed consent.

### 2.2 Liver assessment and phenotypes definitions

Controlled attenuation parameter (CAP) was used to assess hepatic steatosis and LSM to assess fibrosis. Hepatic steatosis was defined as CAP >285 dB/m in the primary analysis, with CAP >263 dB/m used in sensitivity analysis. An LSM threshold of ≥8.0 kPa was used as the operational definition of significant liver fibrosis. These thresholds and the broader use of VCTE for noninvasive steatosis and fibrosis assessment were informed by studies with histologic reference standards and validation/meta-analytic evidence (37–40). In NHANES, a complete VCTE examination (LUAXSTAT = 1) required at least 3 h of fasting, at least 10 valid measurements, and an IQR/median ratio <30%; M- and XL-probe distributions are reported in Supplementary Table S6. In the Chinese clinical study, VCTE was performed after at least 3 h of fasting and required at least 10 valid measurements. Probe selection was based on body habitus at the time of examination; however, the specific probe type was not retained in the available clinical records.

MASLD was operationalized as hepatic steatosis plus at least one cardiometabolic risk factor according to the 2023 multisociety nomenclature consensus and current European and United States guidance (1–3). The five cardiometabolic risk-factor domains were: (1) overweight/central adiposity, defined as BMI ≥ 25 kg/m^2^ in non-Asian participants or ≥23 kg/m^2^ in Asian participants, or increased waist circumference (men ≥94 cm, Asian men ≥90 cm; women ≥80 cm); (2) dysglycemia, defined as fasting glucose ≥100 mg/dL, 2 h post-load glucose ≥140 mg/dL, HbA1c ≥ 5.7%, diagnosed diabetes/prediabetes, or use of glucose-lowering treatment; (3) blood pressure ≥130/85 mmHg or antihypertensive treatment; (4) triglycerides ≥150 mg/dL or lipid-lowering treatment; and (5) low HDL cholesterol or lipid-lowering treatment.

The three-level liver phenotype was defined as follows: G1, no steatosis and no significant liver fibrosis; G2, MASLD without significant fibrosis; and G3, MASLD with significant fibrosis. The four-phenotype sensitivity analysis retained all participants with complete steatosis and significant-fibrosis information and classified them as P1, no steatosis/no significant fibrosis; P2, steatosis/no significant fibrosis; P3, no steatosis/significant fibrosis; and P4, steatosis/significant fibrosis. In NHANES, alcohol intake was estimated from the Alcohol Use Questionnaire (ALQ111, ALQ121, and ALQ130), assuming 14 g of pure alcohol per standard drink. The MASLD definition required alcohol intake <140 g/week in women and <210 g/week in men; participants with unevaluable alcohol exposure or intake at or above these thresholds were excluded.

When age, AST, ALT, and platelet count were available, the Fibrosis-4 (FIB-4) index was calculated as age [years] × aspartate aminotransferase [IU/L]/(platelet [×10^9^/L] × alanine aminotransferase [IU/L]^1/2^) (41). FIB-4 analyses were used as supplementary sensitivity analyses to the VCTE-based definition, with commonly used MASLD risk-stratification thresholds informed by current guidance and validation studies (2, 3, 41–43). In addition, a descriptive assessment of Baveno VII-related candidate classifications was performed in the US fibrosis subgroup (44). Because very few participants met the relevant criteria with LSM ≥ 25 kPa and had GHF events, no formal regression inference for clinically significant portal hypertension (CSPH) was performed.

### 2.3 Glomerular hyperfiltration

Indexed eGFR (mL/min/1.73 m^2^) was calculated from serum creatinine using the 2021 race-free CKD-EPI creatinine equation (31). The primary GHF definition was indexed eGFR strictly above the survey-weighted 95th percentile calculated separately within 10-year age bands and sex in a prespecified low-risk NHANES reference population (Supplementary Table S3). The low-risk reference excluded obesity, known hypertension, dysglycemia, cardiovascular disease, active viral hepatitis, eGFR <60 mL/min/1.73 m^2^, and, when available, albuminuria. The strict “>” rule meant that observations exactly equal to the empirical P95 were not classified as GHF. Threshold-stability diagnostics showed that the 70–79-year reference strata were smallest; these strata were retained to avoid age-based exclusion, and a separate age/sex/BSA-residual analysis was used to test sensitivity to body size and empirical-threshold instability.

To assess transportability, the same fixed NHANES age- and sex-specific P95 thresholds were applied to the Chinese clinical study. Among 416 participants in the prespecified low-risk Chinese calibration reference, 75 (18.0%) exceeded the fixed United States-derived P95, suggesting potential cross-population calibration differences. Therefore, an additional calibration-only sensitivity analysis modeled indexed eGFR as a function of an age spline and sex within the Chinese low-risk reference and defined GHF using the 95th percentile of the residual distribution (10.66 mL/min/1.73 m^2^).

Additional NHANES sensitivity outcomes included indexed eGFR above the age- and sex-specific P90; a residual-based definition in which indexed eGFR was modeled as a function of age, sex, and BSA in the US low-risk reference and GHF was defined as a residual above the survey-weighted P95; and the conventional de-indexed eGFR age- and sex-specific P95 definition.

### 2.4 Covariates and model hierarchy

The covariate hierarchy was designed to distinguish baseline demographic/socioeconomic covariates from body-size factors, exposure-defining cardiometabolic characteristics, and variables that may also lie on shared metabolic pathways. Model 1 adjusted for age and sex; Model 2 additionally adjusted for race/ethnicity, poverty-to-income ratio, education, current smoking, and marital status; Model 3 further included BMI and was designated in the revised analysis as the central body-size-adjusted model because body size was a key methodological concern raised by the original de-indexed outcome; Model 4 additionally included fasting plasma glucose, log-transformed triglycerides, hypertension, and HDL cholesterol as a descriptive metabolic-attenuation model. Model 4 used the fasting subsample and corresponding fasting survey weights. Models 1–4 were repeated within the same fasting subsample to separate covariate attenuation from changes in analytic sample. Given the cross-sectional design and the fact that several metabolic variables contribute to the MASLD definition as well as potentially acting as confounders or intermediates, these models are interpreted as a transparent adjustment hierarchy rather than as identification of a single causal adjustment set.

In the Chinese clinical study, C1 adjusted for age and sex; C2 additionally included current smoking; C3 further included BMI and was designated the central body-size-adjusted model; and C4 additionally included fasting plasma glucose, log-transformed triglycerides, hypertension, and HDL cholesterol. Because these metabolic variables may act as both confounders and components of shared metabolic pathways linking MASLD and GHF, C4/Model 4 was used primarily to describe effect attenuation rather than to support strong causal interpretation.

When medication data were available, sensitivity analyses considered renin-angiotensin system (RAS) inhibitors, diuretics, sodium-glucose cotransporter-2 (SGLT2) inhibitors, and glucagon-like peptide-1 (GLP-1) receptor agonists. Detailed NHANES medication analyses were restricted to the 2017-March 2020 cycle. In the Chinese study, analyses were performed after excluding SGLT2-inhibitor users and after additional adjustment for RAS inhibitors.

### 2.5 Statistical analysis

Continuous variables are summarized as mean ± standard deviation or median (interquartile range), as appropriate, and categorical variables as counts (percentages). NHANES analyses incorporated strata, primary sampling units, and combined-cycle examination weights. For the 2017-March 2020 pre-pandemic component, WTMECPRP was multiplied by 3.2/5.2; for the August 2021–August 2023 component, WTPH2YR was multiplied by 2.0/5.2, after which the rescaled weights were analyzed with the survey design variables. The Chinese clinical study used unweighted analyses. With the primary GHF definition as the binary outcome, multivariable logistic regression estimated ORs and 95% CIs. Adjusted predicted probabilities and risk differences (percentage points, pp) complemented relative effect estimates.

Sensitivity analyses included alternative GHF definitions, multiple imputation (30 imputations) for the NHANES Model 3 covariate analysis, exclusion of active viral hepatitis, medication exclusion/adjustment, the four-phenotype analysis, FIB-4 as an alternative fibrosis marker, and restricted cubic spline (RCS) analyses of CAP and LSM. Missingness and the comparison of Model-3 complete cases with excluded participants are reported in Supplementary Table S7. Because the Chinese G3 group was small, Firth penalized logistic regression was used to assess sparse-data bias. RCS knot locations, reference values, supported exposure ranges, and exposure distributions are provided in Supplementary Table S10 and Supplementary Figure S1; interpretation was restricted to prespecified supported ranges rather than extrapolated into sparse extremes. Blood-pressure stratification and interaction analyses were prespecified as exploratory; when the global interaction test was not significant, differences in stratum-specific ORs were not interpreted as effect modification. All tests were two-sided, with *p* < 0.05 considered statistically significant. All statistical analyses were performed using R software.

## 3 Results

### 3.1 Study populations and baseline characteristics

The primary analysis included 9,140 NHANES participants and 721 participants in the Chinese clinical cohort (Figure 1). In NHANES, G1, G2, and G3 comprised 5,735, 2,703, and 702 participants, respectively; the corresponding numbers in the Chinese cohort were 514, 178, and 29. The three-level analysis included only the prespecified G1/G2/G3 combinations; other steatosis/fibrosis combinations were retained separately in the supplementary four-phenotype analysis.

In NHANES, participants were older and the proportion of men increased from G1 to G3, while obesity, hypertension, diabetes, and other metabolic abnormalities were generally more prominent. Mean BMI was 26.96 ± 5.36, 33.27 ± 6.23, and 38.79 ± 8.11 kg/m^2^ in G1, G2, and G3, respectively, and mean BSA was 1.85 ± 0.22, 2.05 ± 0.24, and 2.19 ± 0.28 m^2^. The prevalence of hypertension increased from 35.4% in G1 to 59.9% in G2 and 75.7% in G3, while diagnosed diabetes was present in 5.2, 16.0, and 34.3%, respectively. CAP and LSM were also higher across the liver phenotypes: median CAP values were 229.0, 320.0, and 340.0 dB/m, and median LSM values were 4.50, 5.30, and 10.50 kPa in G1, G2, and G3. Mean indexed eGFR values were 97.90 ± 19.42, 95.78 ± 20.23, and 94.87 ± 20.99 mL/min/1.73 m^2^, whereas mean de-indexed eGFR values were 104.40 ± 23.09, 113.42 ± 28.22, and 120.57 ± 32.24 mL/min. The prevalence of GHF defined using indexed eGFR was 4.8, 10.1, and 10.5%, respectively (Table 1; Supplementary Table S1).

**Table 1:** Selected baseline characteristics according to liver phenotype in the NHANES study and Chinese clinical replication cohort.

Panel A. NHANES study (*n* = 9,140)				
Characteristic	Overall	G1	G2	G3
Male, *n* (%)	4,300 (49.1%)	2,506 (45.1%)	1,416 (56.0%)	378 (57.1%)
Age, years	46.98 ± 16.31	45.13 ± 16.50	49.98 ± 15.52	51.31 ± 15.01
Current smoking, *n* (%)	1,400 (14.1%)	901 (14.7%)	408 (13.5%)	91 (11.8%)
Body mass index, kg/m^2^	29.64 ± 6.99	26.96 ± 5.36	33.27 ± 6.23	38.79 ± 8.11
Waist circumference, cm	100.07 ± 16.80	92.97 ± 13.20	110.17 ± 13.53	122.80 ± 16.26
Body surface area, m^2^	1.93 ± 0.26	1.85 ± 0.22	2.05 ± 0.24	2.19 ± 0.28
Normal blood pressure, *n* (%)	3,336 (43.3%)	2,527 (52.7%)	698 (29.6%)	111 (16.4%)
Elevated blood pressure, *n* (%)	886 (11.2%)	603 (11.9%)	243 (10.5%)	40 (7.9%)
Hypertension, *n* (%)	4,547 (45.4%)	2,368 (35.4%)	1,648 (59.9%)	531 (75.7%)
Diagnosed diabetes, *n* (%)	1,247 (10.4%)	448 (5.2%)	526 (16.0%)	273 (34.3%)
CAP, dB/m	260.00 (217.00, 307.00)	229.00 (200.00, 257.00)	320.00 (302.00, 351.00)	340.00 (317.00, 378.00)
LSM, kPa	4.90 (4.00, 6.00)	4.50 (3.80, 5.40)	5.30 (4.40, 6.20)	10.50 (9.00, 14.30)
Indexed eGFR, mL/min/1.73 m^2^	97.07 ± 19.81	97.90 ± 19.42	95.78 ± 20.23	94.87 ± 20.99
De-indexed eGFR, mL/min	108.18 ± 25.96	104.40 ± 23.09	113.42 ± 28.22	120.57 ± 32.24
GHF, *n* (%)	698 (6.8%)	322 (4.8%)	293 (10.1%)	83 (10.5%)
Antihypertensive medication use, *n* (%)	2,523 (22.9%)	1,192 (15.5%)	977 (34.0%)	354 (44.5%)

Panel B. Chinese clinical replication cohort (*n* = 721)				
Characteristic	Overall	G1	G2	G3
Male, *n* (%)	426 (59.1%)	297 (57.8%)	117 (65.7%)	12 (41.4%)
Age, years	56.64 ± 11.38	56.58 ± 11.63	56.13 ± 10.57	60.79 ± 11.25
Current smoking, *n* (%)	71 (9.8%)	51 (9.9%)	18 (10.1%)	2 (6.9%)
Body mass index, kg/m^2^	23.46 ± 3.41	22.62 ± 3.25	25.42 ± 2.54	26.31 ± 4.34
Body surface area, m^2^	1.73 ± 0.18	1.70 ± 0.17	1.82 ± 0.16	1.80 ± 0.21
Normal blood pressure, *n* (%)	371 (51.5%)	294 (57.2%)	71 (39.9%)	6 (20.7%)
Elevated blood pressure, *n* (%)	137 (19.0%)	94 (18.3%)	33 (18.5%)	10 (34.5%)
Hypertension, *n* (%)	213 (29.5%)	126 (24.5%)	74 (41.6%)	13 (44.8%)
Diagnosed diabetes, *n* (%)	83 (11.5%)	51 (9.9%)	24 (13.5%)	8 (28.6%)
CAP, dB/m	249.00 (209.00, 292.00)	229.00 (196.25, 254.00)	313.00 (298.00, 331.00)	307.00 (297.00, 337.00)
LSM, kPa	4.40 (3.60, 5.40)	4.20 (3.50, 5.30)	4.60 (3.90, 5.40)	10.20 (8.70, 14.10)
Indexed eGFR, mL/min/1.73 m^2^	97.18 ± 14.84	96.97 ± 15.36	98.15 ± 13.45	95.14 ± 13.56
De-indexed eGFR, mL/min	97.39 ± 18.42	95.09 ± 17.81	103.77 ± 18.33	99.09 ± 20.78
GHF, *n* (%)	127 (17.6%)	93 (18.1%)	26 (14.6%)	8 (27.6%)
Antihypertensive medication use, *n* (%)	154 (22.3%)	93 (18.9%)	52 (30.1%)	9 (34.6%)
SGLT2 inhibitor use, *n* (%)	14 (1.9%)	6 (1.2%)	6 (3.4%)	2 (6.9%)
RAS inhibitor use, *n* (%)	74 (10.3%)	42 (8.2%)	27 (15.2%)	5 (17.2%)
Diuretic use, *n* (%)	18 (2.5%)	8 (1.6%)	8 (4.5%)	2 (6.9%)

G1, no steatosis/no significant fibrosis; G2, MASLD without significant fibrosis; G3, MASLD with significant fibrosis. NHANES percentages and continuous summaries are survey weighted; counts are unweighted. Chinese summaries are unweighted. The main table intentionally prioritizes adiposity/body-size variables (BMI, waist circumference where available, and body surface area), liver measures, kidney measures, and blood-pressure categories. Full sociodemographic, laboratory, and medication characteristics are shown in Supplementary Tables S1 and S2. “No MASLD/no fibrosis” has been replaced throughout by “no steatosis/no significant fibrosis” or the explicit G1 definition.

The Chinese clinical study included 721 participants, with 514, 178, and 29 in G1, G2, and G3, respectively. Mean BMI values were 22.62 ± 3.25, 25.42 ± 2.54, and 26.31 ± 4.34 kg/m^2^, and mean BSA values were 1.70 ± 0.17, 1.82 ± 0.16, and 1.80 ± 0.21 m^2^. The prevalence of hypertension was 24.5, 41.6, and 44.8% across G1, G2, and G3. Mean indexed eGFR values were 96.97 ± 15.36, 98.15 ± 13.45, and 95.14 ± 13.56 mL/min/1.73 m^2^, and the corresponding GHF prevalences were 18.1, 14.6, and 27.6% (Table 1; Supplementary Table S2).

### 3.2 Association of liver phenotype with GHF in NHANES

Stepwise-adjusted logistic regression was used to assess the association between liver phenotype and GHF in NHANES (Table 2; Figure 2). Both G2 and G3 were associated with GHF relative to G1. In the full available-sample analysis, the age- and sex-adjusted ORs were 2.31 (95% CI 1.75–3.06) for G2 versus G1 and 2.47 (95% CI 1.81–3.36) for G3 versus G1. The effect estimates remained broadly stable after additional adjustment for sociodemographic factors (Table 2, Panel A).

**Table 2:** Association of liver phenotype with glomerular hyperfiltration in NHANES, emphasizing body-size and metabolic adjustment.

Panel A. Full-available-sample models								
Model	*N*/events	G2 vs. G1	G2 vs. G1 *p*	G3 vs. G1	G3 vs. G1 *p*	G3 vs. G2	G3 vs. G2 *p*	*p*-trend
Model 1	9140/698	2.31 (1.75–3.06)	<0.001	2.47 (1.81–3.36)	<0.001	1.07 (0.79–1.44)	0.679	<0.001
Model 2	7990/589	2.17 (1.60–2.93)	<0.001	2.39 (1.68–3.39)	<0.001	1.10 (0.76–1.60)	0.611	<0.001
Model 3 (central)	7959/583	2.19 (1.57–3.07)	<0.001	2.46 (1.51–4.00)	<0.001	1.12 (0.74–1.70)	0.596	<0.001

Panel B. Sequential models 1–4 on the same fasting-subsample (directly comparable sample)								
Model	*N*/events	G2 vs. G1	G2 vs. G1 *p*	G3 vs. G1	G3 vs. G1 *p*	G3 vs. G2	G3 vs. G2 *p*	*p*-trend
Model 1	3855/297	2.42 (1.73–3.38)	<0.001	3.14 (2.02–4.89)	<0.001	1.30 (0.86–1.96)	0.212	<0.001
Model 2	3855/297	2.12 (1.48–3.02)	<0.001	2.75 (1.73–4.38)	<0.001	1.30 (0.85–1.99)	0.225	<0.001
Model 3 (+ BMI)	3855/297	2.31 (1.50–3.56)	<0.001	3.18 (1.76–5.73)	<0.001	1.38 (0.89–2.14)	0.153	<0.001
Model 4 (+ metabolic factors)	3855/297	1.94 (1.29–2.93)	0.001	2.08 (1.13–3.83)	0.019	1.07 (0.67–1.71)	0.781	0.006

Panel C. Adjusted probabilities and risk differences in models 3 and 4 on the same fasting-subsample						
Model	G1 adjusted probability	G2 adjusted probability	G3 adjusted probability	G2 vs. G1 RD, pp	G3 vs. G1 RD, pp	G3 vs. G2 RD, pp
Model 3 (+BMI)	4.9% (3.7–6.1)	10.2% (7.5–12.8)	13.2% (8.3–18.2)	+5.3 (+2.3 to +8.3)	+8.4 (+3.0 to +13.7)	+3.1 (−1.5 to +7.6)
Model 4 (+metabolic factors)	5.3% (4.0–6.6)	9.4% (7.3–11.5)	9.9% (6.0–13.9)	+4.1 (+1.5 to +6.7)	+4.6 (+0.2 to +9.0)	+0.5 (−3.3 to +4.4)

The primary outcome is indexed eGFR above the fixed age- and sex-specific NHANES P95 threshold. Model 1 adjusts for age and sex. Model 2 additionally adjusts for race/ethnicity, poverty-income ratio, education, current smoking, and marital status. Model 3 additionally adjusts for BMI and is the central body-size-adjusted model. Model 4 additionally adjusts for fasting plasma glucose, log triglycerides, hypertension, and HDL-C and therefore uses the fasting subsample and fasting survey weights. Panel B uses the same analytic sample for all four models to separate covariate attenuation from sample changes. Risk differences are percentage points (pp). Exact *p*-values are reported.

**Figure 2:**
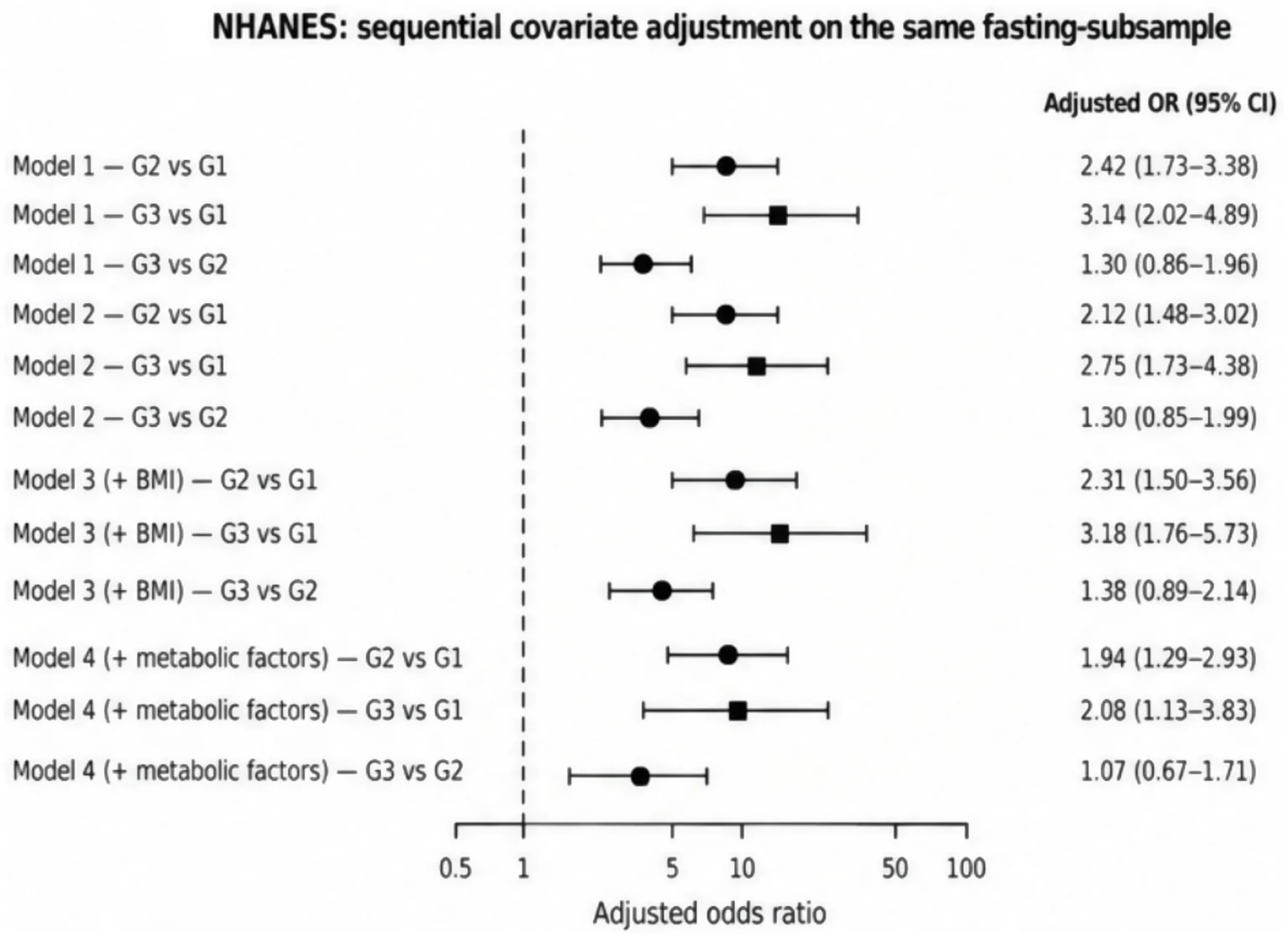
Sequential covariate adjustment of the liver-phenotype–GHF association in NHANES. Odds ratios and 95% confidence intervals are shown for G2 versus G1, G3 versus G1, and G3 versus G2 across Models 1–4 on the identical fasting-subsample. This presentation separates covariate attenuation from changes in analytic sample. Model 3 incorporates BMI; Model 4 additionally incorporates fasting/metabolic variables.

In the identical fasting subsample (n = 3,855; 297 GHF events), Model 1 ORs for G2 versus G1 and G3 versus G1 were 2.42 (95% CI 1.73–3.38) and 3.14 (95% CI 2.02–4.89), respectively. After BMI was added (Model 3), the corresponding ORs were 2.31 (95% CI 1.50–3.56) and 3.18 (95% CI 1.76–5.73). Further adjustment for fasting glucose, triglycerides, hypertension, and HDL cholesterol (Model 4) attenuated the ORs to 1.94 (95% CI 1.29–2.93) and 2.08 (95% CI 1.13–3.83), respectively. G3 did not differ significantly from G2 in either Model 3 or Model 4 (OR 1.38, 95% CI 0.89–2.14; and OR 1.07, 95% CI 0.67–1.71, respectively) (Table 2, Panel B; Figure 2).

In the adjusted probability analysis, Model 3 predicted GHF probabilities were 4.9% (95% CI 3.7–6.1), 10.2% (7.5–12.8), and 13.2% (8.3–18.2) for G1, G2, and G3, respectively. Relative to G1, risk differences were +5.3 pp. (95% CI + 2.3 to +8.3) for G2 and +8.4 pp. (+3.0 to +13.7) for G3; G3 vs. G2 was +3.1 pp. (−1.5 to +7.6). In Model 4, predicted probabilities were 5.3% (4.0–6.6), 9.4% (7.3–11.5), and 9.9% (6.0–13.9), respectively; risk differences were +4.1 pp. (+1.5 to +6.7) for G2 vs. G1, +4.6 pp. (+0.2 to +9.0) for G3 vs. G1, and +0.5 pp. (−3.3 to +4.4) for G3 vs. G2 (Table 2, Panel C).

### 3.3 Replication analysis in the Chinese clinical study

In the Chinese clinical study, the primary C3 analysis using all available participants showed no association between G2 and GHF relative to G1 (OR 1.02, 95% CI 0.60–1.71), whereas the ORs for G3 versus G1 and G3 versus G2 were 3.54 (95% CI 1.39–9.02) and 3.48 (95% CI 1.31–9.22), respectively (Supplementary Table S4). To enable direct comparison with C4 in an identical sample, Table 3 used the common sample of 718 participants. In C3, the ORs for G3 versus G1 and G3 versus G2 were 3.51 (95% CI 1.38–8.93) and 3.47 (95% CI 1.31–9.20), respectively; after further metabolic adjustment in C4, the corresponding ORs were 3.68 (95% CI 1.42–9.54) and 3.17 (95% CI 1.18–8.57). G2 versus G1 remained close to the null in both C3 and C4.

**Table 3:** Independent Chinese clinical study: sequential models, absolute effects, and sparse-data sensitivity.

Panel A. Models C1–C4 on the same C4 sample							
Model	*N*/events	G2 vs. G1	G2 vs. G1 *p*	G3 vs. G1	G3 vs. G1 *p*	G3 vs. G2	G3 vs. G2 *p*
C1	718/127	0.71 (0.44–1.14)	0.154	2.13 (0.89–5.10)	0.091	3.02 (1.17–7.79)	0.023
C2	718/127	0.71 (0.44–1.15)	0.164	2.13 (0.89–5.12)	0.090	3.00 (1.16–7.77)	0.023
C3	718/127	1.01 (0.60–1.70)	0.971	3.51 (1.38–8.93)	0.009	3.47 (1.31–9.20)	0.012
C4	718/127	1.16 (0.67–2.00)	0.598	3.68 (1.42–9.54)	0.007	3.17 (1.18–8.57)	0.023

Panel B. Central C3 adjusted probabilities and risk differences	
Phenotype	Adjusted probability (95% CI)
G1	16.9% (13.8–20.0)
G2	17.1% (11.1–23.1)
G3	39.5% (20.4–58.6)
G2 vs. G1	RD +0.2 (−6.7 to +7.2) pp
G3 vs. G1	RD +22.6 (+3.1 to +42.0) pp
G3 vs. G2	RD +22.4 (+2.6 to +42.1) pp

Panel C. Firth penalized logistic regression sensitivity for the central C3 model		
Contrast	OR (95% CI)	*p*-value
G2 vs. G1	1.02 (0.60–1.70)	0.931
G3 vs. G1	3.60 (1.39–8.81)	0.010
G3 vs. G2	3.51 (1.31–8.96)	0.014

C1 adjusts for age and sex; C2 additionally adjusts for current smoking; C3 additionally adjusts for BMI; C4 additionally adjusts for fasting plasma glucose, log triglycerides, hypertension, and HDL-C. The final G3 group contains 29 participants with 8 GHF events; therefore, fibrosis-specific estimates remain imprecise despite penalized-logistic sensitivity analysis. This table uses “replication” terminology and does not describe the fibrosis-specific result as independently validated.

Adjusted GHF probabilities from C3 were 16.9% for G1, 17.1% for G2, and 39.5% for G3. The risk differences for G3 versus G1 and G3 versus G2 were +22.6 pp. (95% CI + 3.1 to +42.0) and +22.4 pp. (95% CI + 2.6 to +42.1), respectively (Table 3, Panel B). Firth penalized regression yielded similar results: OR 3.60 (95% CI 1.39–8.81) for G3 versus G1 and OR 3.51 (95% CI 1.31–8.96) for G3 versus G2 (Table 3, Panel C).

### 3.4 Sensitivity analyses of GHF definitions and body-size handling

In NHANES, the central findings were relatively robust to alternative GHF definitions. For the age/sex P90 definition, the ORs for G2 versus G1 and G3 versus G1 were 1.87 and 2.17; for the age/sex/BSA-residual P95 definition, they were 2.14 and 2.29; and for the de-indexed eGFR P95 definition, they were 2.06 and 2.10, respectively, all statistically significant. By contrast, the G3-versus-G2 estimates were close to the null and their confidence intervals crossed 1 under all three definitions (Supplementary Table S4; Figure 3). Results after multiple imputation and exclusion of active viral hepatitis were also similar to those from the central Model 3.

**Figure 3:**
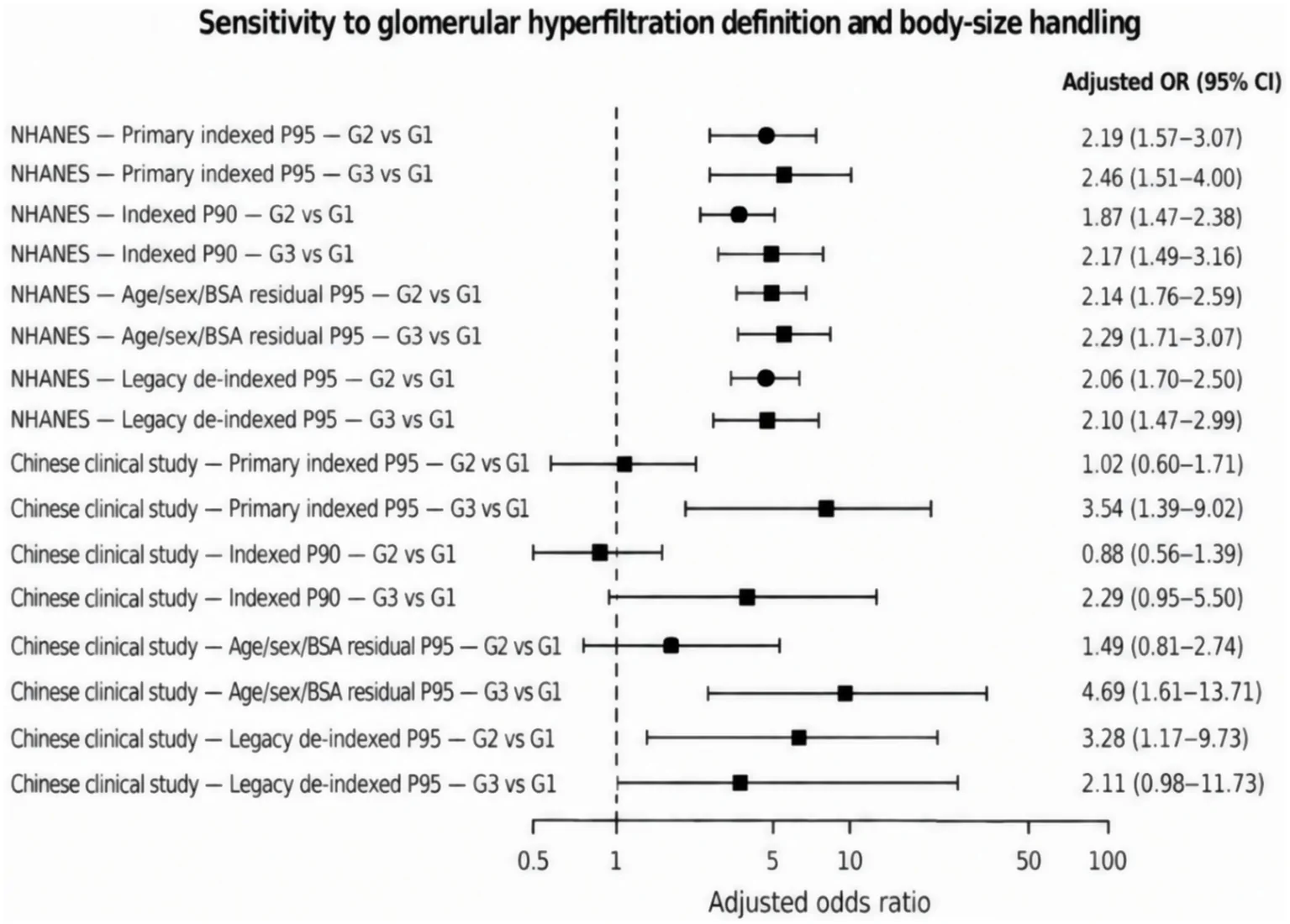
Sensitivity of liver-phenotype associations to GHF definition and body-size handling. The primary outcome is indexed eGFR above the fixed NHANES age- and sex-specific P95. Sensitivity outcomes include indexed P90, an indexed-eGFR residual definition incorporating age, sex, and body surface area, and the legacy de-indexed P95 outcome. Fixed NHANES thresholds/definitions were applied to the Chinese clinical study when transportable.

The Chinese clinical study was more sensitive to outcome definition. Under the primary fixed NHANES indexed P95 definition, the OR for G3 versus G1 was 3.54 (95% CI 1.39–9.02); under the age/sex/BSA-residual P95 definition it was 4.69 (95% CI 1.61–13.71); and under the internally recalibrated Chinese age/sex-residual P95 definition it was 5.07 (95% CI 1.60–16.00). However, under the fixed NHANES P90 definition the 95% CI for G3 versus G1 crossed 1 (OR 2.29, 95% CI 0.95–5.50), while under the de-indexed eGFR P95 definition there were only 22 events and the G3-related estimates were imprecise and not statistically significant.

### 3.5 Continuous associations of CAP and LSM with GHF

RCS analyses showed an overall association between CAP and GHF in NHANES (*p* = 0.000327), with the curve rising at higher CAP values; however, the formal test for nonlinearity was not statistically significant (*p* = 0.124) (Supplementary Figure S1A; Supplementary Table S10). For LSM in NHANES, neither the overall association nor the nonlinearity test was significant (*p* = 0.677 and *p* = 0.531, respectively), providing no evidence of a clear continuous dose–response association between LSM and GHF (Supplementary Figure S1B).

In the Chinese clinical study, CAP was not associated with GHF overall (*p* = 0.677), whereas LSM showed an overall association with GHF (*p* = 0.023) and some evidence of nonlinearity (*p* = 0.046). After simultaneous conditioning on CAP, the overall association of LSM remained significant (*p* = 0.023) (Supplementary Figures S1C,D; Supplementary Table S10).

### 3.6 Exploratory analyses by blood-pressure status

After stratification of NHANES participants into normal blood pressure, elevated blood pressure, and hypertension, some stratum-specific ORs differed numerically. The OR for G2 versus G1 was >1 in all three blood-pressure subgroups. Point estimates for G3 versus G1 were also >1 across subgroups, although some 95% CIs crossed 1; G3 versus G2 did not show a consistent direction. The global liver-phenotype × blood-pressure-category interaction was not significant (*p* = 0.477). These subgroup findings should therefore be regarded as exploratory and should not be interpreted as evidence of effect modification by blood-pressure status (Supplementary Figure S2; Supplementary Table S12).

### 3.7 Other phenotype, medication, and missing-data analyses

After 30 multiple imputations in NHANES, Model 3 estimates were nearly identical to the complete-case estimates (G2 vs. G1 OR 2.20; G3 vs. G1 OR 2.52; G3 vs. G2 OR 1.14), suggesting that the main findings were unlikely to be explained entirely by missing Model 3 covariates. In most analyses excluding users of SGLT2 inhibitors, GLP-1 receptor agonists, RAS inhibitors, or diuretics, the positive associations of G2/G3 relative to G1 were retained. In the Chinese study, the G3-related signal also persisted after exclusion of SGLT2-inhibitor users or after additional adjustment for RAS inhibitor use (Supplementary Tables S7, S8).

Fibrosis-specific sensitivity analyses showed clear cross-study differences. Among NHANES participants with MASLD, the central Model 3 OR for significant fibrosis versus no significant fibrosis was 1.14 (95% CI 0.73–1.79) and remained non-significant after additional control for CAP using either a linear or spline specification. By contrast, the corresponding OR was approximately 3.9 in the Chinese MASLD subgroup, although the confidence interval was wide (Supplementary Table S9). Results of the FIB-4 sensitivity analyses were also not consistent across all metrics (Supplementary Table S11). In the CAP-conditioned NHANES model, the weighted correlation between CAP and the significant-fibrosis indicator was 0.202 and adjusted GVIF values were close to 1, arguing against problematic collinearity (Supplementary Table S9).

## 4 Discussion

Using a large population-based sample together with an independent clinical study, we systematically evaluated the relationships of MASLD, liver fibrosis, and GHF. First, in the nationally representative NHANES sample, both G2 and G3 were associated with higher odds and higher adjusted absolute probabilities of GHF relative to G1. These associations persisted after BMI adjustment but were attenuated after further inclusion of fasting glucose, triglycerides, hypertension, and HDL cholesterol. Second, across the central NHANES model, sequential models in the identical fasting subsample, and multiple GHF definitions, G3 did not differ clearly from G2. Thus, the US data support an association between MASLD phenotypes and GHF but do not support a monotonic pattern in which significant fibrosis necessarily confers additional GHF beyond MASLD itself. Third, the Chinese clinical study showed a different pattern: G2 was similar to G1, whereas G3 had higher odds of GHF than both G1 and G2. However, G3 included only 29 participants and 8 events, and results were not stable across all alternative GHF definitions. Accordingly, the Chinese findings are more appropriately interpreted as an independent replication signal for the G3 hyperfiltration phenotype rather than full external validation of a fibrosis-specific effect.

These findings are informative when considered alongside, but not as fully concordant with, the existing literature. Previous studies have consistently linked NAFLD/MASLD with an increased risk of CKD (9–11, 13, 14, 24–26), whereas evidence regarding GHF as an earlier functional phenotype remains limited. Abbate et al. reported an association between NAFLD and GHF in adults with metabolic syndrome (21). In adults with MASLD, Dalbeni et al. (22) reported an association between fibrosis assessed by VCTE and GHF, while the Korean longitudinal cohort suggested that GHF may precede subsequent NAFLD and fibrosis progression (23). Contemporary mechanistic frameworks for NAFLD/MASLD emphasize insulin resistance, lipid overload, adipose-tissue dysfunction, inflammation, oxidative stress, cardiovascular-metabolic interactions, and inter-organ signaling (36, 45–50), while fibrosis stage is a major prognostic determinant of long-term outcomes in NAFLD (51). In contrast, our study used age- and sex-specific P95 thresholds and explicitly addressed body-size handling in NHANES, but did not identify an incremental fibrosis effect within the US MASLD subgroup. Differences in GHF thresholds, fibrosis thresholds, referral spectrum, adiposity, age distribution, and model specification may partly explain the discrepant findings and indicate that “hyperfiltration” is not necessarily the same statistical or physiological phenotype across studies.

Handling of body size is one of the most important methodological features of this study. In NHANES, mean indexed eGFR decreased slightly from G1 to G3, whereas mean de-indexed eGFR increased from 104.40 to 120.57 mL/min, in parallel with an increase in BSA from 1.85 to 2.19 m^2^. This opposite pattern illustrates how de-indexed eGFR can increase mechanically as BSA increases (27, 28). Consequently, if GHF is identified solely from de-indexed eGFR, individuals with MASLD and greater adiposity may be more likely to be classified as hyperfiltering. We therefore used the age- and sex-specific P95 of indexed eGFR as the primary outcome and additionally applied an age/sex/BSA-residual definition. The associations of G2 and G3 with GHF relative to G1 remained in NHANES under these approaches, arguing against simple mathematical amplification by BSA as the sole explanation for the primary findings. Nonetheless, this does not eliminate body-composition-related bias, because creatinine is influenced by muscle mass and body composition, and estimation error may be greater at high eGFR levels (27, 31–33).

For liver fibrosis, our findings favor population heterogeneity rather than a uniform dose–response relationship. In NHANES, G3 did not differ significantly from G2, the significant-fibrosis contrast within MASLD remained close to the null after adjustment for CAP, and continuous LSM was not associated with GHF overall. In contrast, both G3 contrasts and continuous LSM analyses showed positive signals in the Chinese clinical study. The Chinese cohort consisted of single-center hospitalized patients whose disease spectrum, body size, and baseline GHF prevalence differed substantially from those in NHANES; moreover, the G3 subgroup was very small. These factors may amplify or alter fibrosis-related estimates. FIB-4 sensitivity analyses likewise did not show a uniform pattern of progressively greater GHF with increasing fibrosis burden. Thus, the available data are insufficient to establish significant fibrosis as a determinant of GHF independent of MASLD and metabolic status; a more cautious interpretation is that the fibrosis-related hyperfiltration signal observed in the Chinese clinical sample warrants further validation.

Biologically, MASLD and GHF may share multiple metabolic and hemodynamic pathways rather than being linked through a single mechanism in which liver disease directly causes renal hyperfiltration. Obesity, insulin resistance, hyperglycemia, hypertension, and dyslipidemia can alter glomerular hemodynamics, while activation of the renin-angiotensin system, oxidative stress, inflammation, adipose-tissue dysfunction, and inter-organ signaling may contribute to both hepatic and renal changes (12, 14–17, 45–48, 50). Cardiovascular and autonomic abnormalities may also be relevant: a recent study in MASLD reported markedly impaired cardiorespiratory fitness and a high prevalence of chronotropic incompetence in advanced fibrosis, suggesting a possible autonomic/hemodynamic component (52). This should be regarded as a mechanistic hypothesis rather than evidence of causality in the present study. In NHANES, further adjustment for fasting glucose, triglycerides, hypertension, and HDL cholesterol attenuated the G2/G3 associations relative to G1, consistent with a substantial contribution from shared metabolic burden. Because these variables are related to the MASLD definition and may also lie on shared pathways, Model 4 is interpreted as descriptive attenuation rather than a test of an ‘independent liver effect’.

The blood-pressure analyses should likewise be interpreted cautiously. Although some stratum-specific ORs differed numerically, the global interaction *p*-value was 0.477 in NHANES, and the conventional interaction analysis in the Chinese study was limited by sparse G3 cells. The present data therefore provide insufficient evidence that blood-pressure status modifies the association between liver phenotype and GHF, while not establishing that the association is identical across blood-pressure strata. Treating blood pressure as a potential shared metabolic/hemodynamic factor is more consistent with the data than inferring effect modification from stratum-specific point estimates.

GHF in this study is best regarded as a research phenotype representing the upper tail of estimated renal filtration, not as a diagnostic label or demonstrated renal injury. Definitions of GHF remain heterogeneous (18), and the Korean longitudinal study suggesting that GHF may precede NAFLD/fibrosis progression (23) does not establish the temporal direction in our data. Accordingly, we describe GHF only as a potential early renal functional phenotype. Prospective studies should incorporate measured GFR or validated alternative filtration markers, urinary albumin, metabolic exposures, and longitudinal liver-fibrosis assessment to determine whether persistent GHF predicts subsequent CKD, albuminuria, or eGFR decline.

This study has several strengths. First, it combined nationally representative NHANES data incorporating the complex survey design with an independent Chinese clinical study and used a prespecified three-level liver phenotype and primary GHF framework. Second, hepatic steatosis and fibrosis were assessed using VCTE measures rather than liver enzymes alone. Third, the study addressed body-size handling through complementary analyses of indexed eGFR, BMI adjustment, an age/sex/BSA-residual approach, and de-indexed eGFR, while also reporting adjusted probabilities and risk differences. Fourth, the use of sequential Models 1–4 in the identical fasting subsample, multiple imputation, medication sensitivity analyses, and Firth regression improved transparency regarding sample changes and sparse-data limitations.

Several limitations should be acknowledged. First, the cross-sectional design cannot establish temporal sequence or causality. Second, kidney function was estimated from serum creatinine rather than measured GFR; creatinine is influenced by muscle mass, diet, frailty, and liver disease, and estimation error may be greater at high eGFR levels (27, 31–33). A harmonized cystatin-C analysis was not available in the supplied cross-cohort analytic dataset and should not be inferred from the present results. Third, GHF lacks an internationally standardized definition (18). Notably, 18.0% of the adapted low-risk Chinese reference exceeded the fixed NHANES P95, indicating cross-population/laboratory calibration differences; although the G3 signal persisted under internal residual recalibration, direct comparison of absolute GHF prevalence remains limited. Finally, missingness was nontrivial for some variables in the Chinese source dataset (particularly HbA1c), limiting analyses that rely on these measures.

## 5 Conclusion

In NHANES, MASLD without significant fibrosis and MASLD with significant fibrosis were each associated with GHF defined from age- and sex-specific indexed eGFR after central BMI adjustment, but the associations were attenuated after further metabolic adjustment and there was no clear evidence that significant fibrosis added to MASLD (G3 vs. G2). In the Chinese clinical study, G3 showed higher odds than G1 and G2 whereas G2 was similar to G1, but this result is limited by sparse data and cross-population threshold calibration. Overall, the findings support an association between MASLD/metabolic burden and GHF, while fibrosis-specific effects were not consistent across populations. Prospective studies using measured GFR are needed to determine the temporal and prognostic significance of GHF in MASLD.

## Data Availability

The raw data supporting the conclusions of this article will be made available by the authors, without undue reservation.
