# Supplementary material for: Associations of metabolic dysfunction-associated steatotic liver disease and significant fibrosis phenotypes with glomerular hyperfiltration defined by indexed eGFR
Source: Front Nutr. 2026 Sep 30;13:1944355. doi: 10.3389/fnut.2026.1944355 (PMC13661452; doi:10.3389/fnut.2026.1944355)
Supplement: Supplementary file 1 [file Supplementary_file_1.DOCX]

Supplementary Material

# 1 Supplementary Figures and Tables

## 1.1 Supplementary Tables

**Supplementary Table S1. Full baseline characteristics according to liver phenotype in the NHANES study.**

| **Characteristic** | **Overall** | **G1** | **G2** | **G3** | **P value** |
| --- | --- | --- | --- | --- | --- |
| Sex, n (%) |  |  |  |  | <0.001 |
| Male | 4300 (49.1%) | 2506 (45.1%) | 1416 (56.0%) | 378 (57.1%) | — |
| Female | 4840 (50.9%) | 3229 (54.9%) | 1287 (44.0%) | 324 (42.9%) | — |
| Age, years | 46.98 ± 16.31 | 45.13 ± 16.50 | 49.98 ± 15.52 | 51.31 ± 15.01 | <0.001 |
| Race/ethnicity, n (%) |  |  |  |  | <0.001 |
| Mexican American | 983 (8.4%) | 496 (6.9%) | 399 (11.5%) | 88 (9.6%) | — |
| Non-Hispanic Asian | 891 (5.9%) | 594 (6.2%) | 252 (5.7%) | 45 (3.9%) | — |
| Non-Hispanic Black | 1805 (10.4%) | 1243 (11.6%) | 446 (8.7%) | 116 (7.7%) | — |
| Non-Hispanic White | 3973 (62.2%) | 2455 (62.0%) | 1165 (61.1%) | 353 (67.7%) | — |
| Other / Multiracial | 489 (4.6%) | 315 (4.6%) | 143 (4.7%) | 31 (3.8%) | — |
| Other Hispanic | 999 (8.5%) | 632 (8.8%) | 298 (8.4%) | 69 (7.3%) | — |
| Education level, n (%) |  |  |  |  | <0.001 |
| College or above | 5867 (65.4%) | 3810 (68.2%) | 1641 (61.7%) | 416 (55.1%) | — |
| High school or equivalent | 1980 (25.6%) | 1181 (23.4%) | 625 (28.4%) | 174 (33.9%) | — |
| Less than high school | 1287 (9.0%) | 741 (8.4%) | 434 (9.9%) | 112 (11.0%) | — |
| Poverty-income ratio | 3.40 (1.74, 5.00) | 3.49 (1.75, 5.00) | 3.28 (1.74, 5.00) | 2.98 (1.74, 5.00) | 0.047 |
| Marital status, n (%) |  |  |  |  | <0.001 |
| Married/Living with partner | 5361 (63.2%) | 3236 (60.7%) | 1689 (68.3%) | 436 (65.9%) | — |
| Never married | 1845 (20.1%) | 1322 (23.0%) | 426 (15.2%) | 97 (14.3%) | — |
| Widowed/Divorced/Separated | 1926 (16.6%) | 1171 (16.3%) | 586 (16.5%) | 169 (19.8%) | — |
| Current smoking, n (%) |  |  |  |  | 0.187 |
| Yes | 1400 (14.1%) | 901 (14.7%) | 408 (13.5%) | 91 (11.8%) | — |
| No | 7733 (85.9%) | 4829 (85.3%) | 2293 (86.5%) | 611 (88.2%) | — |
| Alcohol intake, g/week | 6.5 (0.4, 28.0) | 8.1 (0.4, 32.3) | 4.8 (0.4, 28.0) | 2.4 (0.0, 16.2) | 0.021 |
| Body mass index, kg/m² | 29.64 ± 6.99 | 26.96 ± 5.36 | 33.27 ± 6.23 | 38.79 ± 8.11 | <0.001 |
| BMI category, n (%) |  |  |  |  | <0.001 |
| Underweight (<18.5) | 109 (1.3%) | 108 (2.1%) | 1 (0.0%) | 0 (0.0%) | — |
| Normal weight (18.5 to <25) | 2191 (24.9%) | 2041 (36.8%) | 138 (4.7%) | 12 (1.2%) | — |
| Overweight (25 to <30) | 2978 (32.7%) | 2084 (37.1%) | 803 (28.2%) | 91 (11.4%) | — |
| Obesity (>=30) | 3828 (41.1%) | 1479 (24.0%) | 1756 (67.1%) | 593 (87.3%) | — |
| Waist circumference, cm | 100.07 ± 16.80 | 92.97 ± 13.20 | 110.17 ± 13.53 | 122.80 ± 16.26 | <0.001 |
| Body surface area, m² | 1.93 ± 0.26 | 1.85 ± 0.22 | 2.05 ± 0.24 | 2.19 ± 0.28 | <0.001 |
| Systolic blood pressure, mmHg | 120.33 ± 16.03 | 118.43 ± 15.71 | 123.55 ± 16.23 | 124.29 ± 15.27 | <0.001 |
| Diastolic blood pressure, mmHg | 74.20 ± 10.36 | 72.64 ± 9.92 | 76.77 ± 10.43 | 77.76 ± 10.94 | <0.001 |
| Blood pressure category, n (%) |  |  |  |  | <0.001 |
| Normal | 3336 (43.3%) | 2527 (52.7%) | 698 (29.6%) | 111 (16.4%) | — |
| Elevated | 886 (11.2%) | 603 (11.9%) | 243 (10.5%) | 40 (7.9%) | — |
| Hypertension | 4547 (45.4%) | 2368 (35.4%) | 1648 (59.9%) | 531 (75.7%) | — |
| Diagnosed diabetes, n (%) |  |  |  |  | <0.001 |
| Yes | 1247 (10.4%) | 448 (5.2%) | 526 (16.0%) | 273 (34.3%) | — |
| No | 7890 (89.6%) | 5284 (94.8%) | 2177 (84.0%) | 429 (65.7%) | — |
| Fasting plasma glucose, mg/dL | 108.92 ± 32.61 | 102.46 ± 22.88 | 117.44 ± 40.45 | 134.95 ± 49.43 | <0.001 |
| HbA1c, % | 5.67 ± 0.98 | 5.46 ± 0.70 | 5.93 ± 1.17 | 6.41 ± 1.44 | <0.001 |
| Total cholesterol, mg/dL | 187.99 ± 40.54 | 186.93 ± 39.77 | 191.52 ± 41.19 | 183.50 ± 43.48 | <0.001 |
| LDL-C, mg/dL | 110.86 ± 34.96 | 109.91 ± 33.70 | 114.15 ± 36.49 | 105.57 ± 39.12 | 0.012 |
| HDL-C, mg/dL | 53.05 ± 14.81 | 56.47 ± 15.04 | 47.43 ± 12.31 | 45.23 ± 11.80 | <0.001 |
| Fasting triglycerides, mg/dL | 86.00 (58.26, 131.00) | 72.00 (51.41, 107.00) | 116.97 (83.00, 164.92) | 120.00 (87.62, 173.00) | <0.001 |
| ALT, U/L | 18.00 (13.00, 27.00) | 16.00 (12.00, 23.00) | 22.00 (16.00, 31.00) | 28.00 (19.00, 40.00) | <0.001 |
| AST, U/L | 19.00 (16.00, 24.00) | 19.00 (16.00, 23.00) | 20.00 (16.00, 25.00) | 22.00 (18.00, 31.00) | <0.001 |
| GGT, U/L | 20.00 (13.00, 30.00) | 17.00 (12.00, 25.00) | 24.00 (18.00, 35.00) | 31.00 (21.00, 52.00) | <0.001 |
| Albumin, g/dL | 4.12 ± 0.33 | 4.15 ± 0.32 | 4.09 ± 0.32 | 4.02 ± 0.34 | <0.001 |
| C-reactive protein, mg/L | 1.73 (0.78, 3.96) | 1.27 (0.61, 2.96) | 2.68 (1.26, 5.57) | 4.06 (1.97, 7.85) | <0.001 |
| Serum creatinine, mg/dL | 0.84 (0.72, 0.99) | 0.84 (0.72, 0.97) | 0.85 (0.72, 0.99) | 0.87 (0.70, 1.01) | 0.290 |
| Uric acid, mg/dL | 5.20 (4.30, 6.10) | 4.90 (4.00, 5.80) | 5.60 (4.80, 6.60) | 5.90 (4.90, 6.90) | <0.001 |
| Urinary albumin-to-creatinine ratio, mg/g | 6.71 (4.52, 11.60) | 6.38 (4.38, 10.44) | 7.07 (4.66, 13.00) | 10.00 (5.54, 23.81) | 0.048 |
| Controlled attenuation parameter, dB/m | 260.00 (217.00, 307.00) | 229.00 (200.00, 257.00) | 320.00 (302.00, 351.00) | 340.00 (317.00, 378.00) | <0.001 |
| Liver stiffness measurement, kPa | 4.90 (4.00, 6.00) | 4.50 (3.80, 5.40) | 5.30 (4.40, 6.20) | 10.50 (9.00, 14.30) | <0.001 |
| Indexed eGFR, mL/min/1.73 m² | 97.07 ± 19.81 | 97.90 ± 19.42 | 95.78 ± 20.23 | 94.87 ± 20.99 | 0.001 |
| De-indexed eGFR, mL/min | 108.18 ± 25.96 | 104.40 ± 23.09 | 113.42 ± 28.22 | 120.57 ± 32.24 | <0.001 |
| Glomerular hyperfiltration, n (%) |  |  |  |  | <0.001 |
| Yes | 698 (6.8%) | 322 (4.8%) | 293 (10.1%) | 83 (10.5%) | — |
| No | 8442 (93.2%) | 5413 (95.2%) | 2410 (89.9%) | 619 (89.5%) | — |
| Antihypertensive medication use, n (%) |  |  |  |  | <0.001 |
| Yes | 2523 (22.9%) | 1192 (15.5%) | 977 (34.0%) | 354 (44.5%) | — |
| No | 6426 (77.1%) | 4434 (84.5%) | 1656 (66.0%) | 336 (55.5%) | — |
| Lipid-lowering medication use, n (%) |  |  |  |  | <0.001 |
| Yes | 2067 (20.6%) | 1020 (15.7%) | 801 (28.8%) | 246 (30.0%) | — |
| No | 6426 (79.4%) | 4315 (84.3%) | 1699 (71.2%) | 412 (70.0%) | — |
| NHANES study cycle, n (%) |  |  |  |  | 0.158 |
| 2017-March 2020 | 5638 (63.9%) | 3478 (63.0%) | 1744 (66.4%) | 416 (62.3%) | — |
| August 2021-August 2023 | 3502 (36.1%) | 2257 (37.0%) | 959 (33.6%) | 286 (37.7%) | — |

NHANES values are unweighted n (survey-weighted %) or survey-weighted summary statistics. P values account for the complex survey design.

**Supplementary Table S2. Full baseline characteristics according to liver phenotype in the Chinese clinical study.**

| **Characteristic** | **Overall** | **G1** | **G2** | **G3** | **P value** |
| --- | --- | --- | --- | --- | --- |
| Sex, n (%) |  |  |  |  | 0.025 |
| Male | 426 (59.1%) | 297 (57.8%) | 117 (65.7%) | 12 (41.4%) | — |
| Female | 295 (40.9%) | 217 (42.2%) | 61 (34.3%) | 17 (58.6%) | — |
| Age, years | 56.64 ± 11.38 | 56.58 ± 11.63 | 56.13 ± 10.57 | 60.79 ± 11.25 | 0.120 |
| Ethnicity, n (%) |  |  |  |  | 0.205 |
| Han ethnicity | 707 (98.1%) | 502 (97.7%) | 177 (99.4%) | 28 (96.6%) | — |
| Ethnic minorities | 14 (1.9%) | 12 (2.3%) | 1 (0.6%) | 1 (3.4%) | — |
| Marital status, n (%) |  |  |  |  | 0.288 |
| Widowed | 1 (0.1%) | 0 (0.0%) | 1 (0.6%) | 0 (0.0%) | — |
| Never married | 3 (0.4%) | 3 (0.6%) | 0 (0.0%) | 0 (0.0%) | — |
| Married | 717 (99.4%) | 511 (99.4%) | 177 (99.4%) | 29 (100.0%) | — |
| Current smoking, n (%) |  |  |  |  | 0.972 |
| Yes | 71 (9.8%) | 51 (9.9%) | 18 (10.1%) | 2 (6.9%) | — |
| No | 650 (90.2%) | 463 (90.1%) | 160 (89.9%) | 27 (93.1%) | — |
| Current alcohol use, n (%) |  |  |  |  | 0.007 |
| Yes | 60 (8.3%) | 33 (6.4%) | 25 (14.0%) | 2 (6.9%) | — |
| No | 661 (91.7%) | 481 (93.6%) | 153 (86.0%) | 27 (93.1%) | — |
| Body mass index, kg/m² | 23.46 ± 3.41 | 22.62 ± 3.25 | 25.42 ± 2.54 | 26.31 ± 4.34 | <0.001 |
| BMI category, n (%) |  |  |  |  | <0.001 |
| Underweight (<18.5) | 55 (7.6%) | 54 (10.5%) | 0 (0.0%) | 1 (3.4%) | — |
| Normal/low BMI (<23) | 260 (36.1%) | 225 (43.8%) | 29 (16.3%) | 6 (20.7%) | — |
| Asian adiposity threshold (23 to <25) | 170 (23.6%) | 115 (22.4%) | 50 (28.1%) | 5 (17.2%) | — |
| Overweight (25 to <30) | 217 (30.1%) | 113 (22.0%) | 92 (51.7%) | 12 (41.4%) | — |
| Obesity (>=30) | 19 (2.6%) | 7 (1.4%) | 7 (3.9%) | 5 (17.2%) | — |
| Body surface area, m² | 1.73 ± 0.18 | 1.70 ± 0.17 | 1.82 ± 0.16 | 1.80 ± 0.21 | <0.001 |
| Systolic blood pressure, mmHg | 118.66 ± 9.94 | 117.66 ± 9.03 | 120.94 ± 11.81 | 122.38 ± 9.90 | <0.001 |
| Diastolic blood pressure, mmHg | 71.09 ± 7.46 | 70.49 ± 6.89 | 72.66 ± 8.61 | 72.17 ± 8.24 | 0.003 |
| Blood pressure category, n (%) |  |  |  |  | <0.001 |
| Normal | 371 (51.5%) | 294 (57.2%) | 71 (39.9%) | 6 (20.7%) | — |
| Elevated | 137 (19.0%) | 94 (18.3%) | 33 (18.5%) | 10 (34.5%) | — |
| Hypertension | 213 (29.5%) | 126 (24.5%) | 74 (41.6%) | 13 (44.8%) | — |
| Diagnosed diabetes, n (%) |  |  |  |  | 0.011 |
| Yes | 83 (11.5%) | 51 (9.9%) | 24 (13.5%) | 8 (28.6%) | — |
| No | 637 (88.5%) | 463 (90.1%) | 154 (86.5%) | 20 (71.4%) | — |
| Fasting plasma glucose, mmol/L | 5.44 ± 1.71 | 5.40 ± 1.78 | 5.53 ± 1.51 | 5.64 ± 1.75 | 0.548 |
| HbA1c, % | 6.24 ± 1.48 | 6.23 ± 1.64 | 6.21 ± 1.17 | 6.64 ± 1.10 | 0.765 |
| Total cholesterol, mmol/L | 4.45 ± 1.05 | 4.40 ± 1.06 | 4.62 ± 1.00 | 4.38 ± 1.05 | 0.050 |
| LDL-C, mmol/L | 2.90 ± 0.79 | 2.84 ± 0.79 | 3.08 ± 0.74 | 2.84 ± 0.81 | 0.001 |
| HDL-C, mmol/L | 1.20 ± 0.32 | 1.22 ± 0.34 | 1.12 ± 0.24 | 1.13 ± 0.35 | <0.001 |
| Triglycerides, mmol/L | 1.37 (1.00, 1.91) | 1.23 (0.92, 1.68) | 1.85 (1.38, 2.50) | 1.46 (1.05, 2.34) | <0.001 |
| ALT, U/L | 20.00 (14.00, 31.00) | 18.00 (13.00, 28.00) | 25.00 (18.00, 36.00) | 29.00 (23.00, 34.00) | <0.001 |
| AST, U/L | 22.00 (18.00, 28.00) | 21.00 (17.00, 27.00) | 24.00 (19.00, 28.00) | 32.00 (25.00, 49.00) | <0.001 |
| GGT, U/L | 23.60 (16.50, 37.92) | 20.70 (15.10, 31.80) | 29.75 (21.72, 45.75) | 45.10 (32.10, 89.40) | <0.001 |
| Albumin, g/L | 45.52 ± 4.66 | 45.24 ± 4.41 | 46.57 ± 4.95 | 44.19 ± 6.09 | 0.001 |
| C-reactive protein | 1.21 (0.43, 3.00) | 1.08 (0.37, 3.04) | 1.56 (0.63, 2.75) | 1.99 (1.21, 4.86) | 0.003 |
| Urea | 5.49 (4.52, 6.57) | 5.46 (4.47, 6.53) | 5.56 (4.78, 6.76) | 5.55 (4.48, 6.58) | 0.363 |
| Serum creatinine, µmol/L | 69.40 (60.40, 79.90) | 69.30 (60.00, 80.30) | 70.10 (62.12, 79.58) | 65.40 (57.20, 76.10) | 0.216 |
| Uric acid, µmol/L | 317.00 (262.50, 381.00) | 302.00 (250.75, 362.00) | 360.50 (304.00, 413.00) | 337.00 (289.00, 425.00) | <0.001 |
| Uric acid-to-creatinine ratio | 77.00 (65.00, 94.00) | 76.00 (64.00, 93.00) | 79.00 (65.00, 96.00) | 80.00 (73.00, 102.00) | 0.160 |
| Controlled attenuation parameter, dB/m | 249.00 (209.00, 292.00) | 229.00 (196.25, 254.00) | 313.00 (298.00, 331.00) | 307.00 (297.00, 337.00) | <0.001 |
| Liver stiffness measurement, kPa | 4.40 (3.60, 5.40) | 4.20 (3.50, 5.30) | 4.60 (3.90, 5.40) | 10.20 (8.70, 14.10) | <0.001 |
| Indexed eGFR, mL/min/1.73 m² | 97.18 ± 14.84 | 96.97 ± 15.36 | 98.15 ± 13.45 | 95.14 ± 13.56 | 0.495 |
| De-indexed eGFR, mL/min | 97.39 ± 18.42 | 95.09 ± 17.81 | 103.77 ± 18.33 | 99.09 ± 20.78 | <0.001 |
| Glomerular hyperfiltration, n (%) |  |  |  |  | 0.204 |
| Yes | 127 (17.6%) | 93 (18.1%) | 26 (14.6%) | 8 (27.6%) | — |
| No | 594 (82.4%) | 421 (81.9%) | 152 (85.4%) | 21 (72.4%) | — |
| Anti-hypertensive medication use, n (%) |  |  |  |  | 0.003 |
| Yes | 154 (22.3%) | 93 (18.9%) | 52 (30.1%) | 9 (34.6%) | — |
| No | 538 (77.7%) | 400 (81.1%) | 121 (69.9%) | 17 (65.4%) | — |
| Glucose-lowering medication use, n (%) |  |  |  |  | 0.007 |
| Yes | 70 (9.8%) | 43 (8.4%) | 19 (10.9%) | 8 (27.6%) | — |
| No | 642 (90.2%) | 466 (91.6%) | 155 (89.1%) | 21 (72.4%) | — |
| Lipid-lowering medication use, n (%) |  |  |  |  | 0.065 |
| Yes | 35 (4.9%) | 25 (4.9%) | 6 (3.4%) | 4 (13.8%) | — |
| No | 686 (95.1%) | 489 (95.1%) | 172 (96.6%) | 25 (86.2%) | — |
| SGLT2 inhibitor use, n (%) |  |  |  |  | 0.027 |
| Yes | 14 (1.9%) | 6 (1.2%) | 6 (3.4%) | 2 (6.9%) | — |
| No | 707 (98.1%) | 508 (98.8%) | 172 (96.6%) | 27 (93.1%) | — |
| RAS inhibitor use, n (%) |  |  |  |  | 0.011 |
| Yes | 74 (10.3%) | 42 (8.2%) | 27 (15.2%) | 5 (17.2%) | — |
| No | 647 (89.7%) | 472 (91.8%) | 151 (84.8%) | 24 (82.8%) | — |
| Diuretic use, n (%) |  |  |  |  | 0.024 |
| Yes | 18 (2.5%) | 8 (1.6%) | 8 (4.5%) | 2 (6.9%) | — |
| No | 703 (97.5%) | 506 (98.4%) | 170 (95.5%) | 27 (93.1%) | — |

Values are n (%) or mean ± SD/median (interquartile range), as appropriate. HbA1c and other incompletely measured variables should be interpreted according to the missingness table.

**Supplementary Table S3. Age- and sex-specific eGFR thresholds used to define glomerular hyperfiltration.**

| **Sex** | **Age, years** | **Healthy reference n** | **Indexed eGFR P90** | **Indexed eGFR P95 (primary)** | **De-indexed eGFR P95** |
| --- | --- | --- | --- | --- | --- |
| Male | 20-29 | 354 | 127.9 | 129.8 | 148.1 |
| Male | 30-39 | 297 | 119.7 | 120.8 | 145.9 |
| Male | 40-49 | 196 | 112.8 | 114.0 | 135.1 |
| Male | 50-59 | 107 | 104.8 | 106.6 | 124.4 |
| Male | 60-69 | 104 | 99.1 | 100.8 | 118.4 |
| Male | 70-79 | 44 | 91.6 | 93.5 | 103.9 |
| Female | 20-29 | 384 | 129.6 | 130.9 | 132.7 |
| Female | 30-39 | 365 | 122.0 | 123.8 | 129.7 |
| Female | 40-49 | 264 | 114.7 | 115.8 | 119.2 |
| Female | 50-59 | 165 | 106.9 | 108.2 | 110.5 |
| Female | 60-69 | 148 | 100.5 | 101.4 | 106.4 |
| Female | 70-79 | 48 | 98.5 | 102.9 | 100.5 |

Primary indexed-eGFR P95 thresholds were derived once from the pooled NHANES low-risk reference using combined survey weights and 10-year age bands, then applied unchanged to the Chinese cohort. The low-risk reference excluded obesity and known hypertension, dysglycemia, cardiovascular disease, active viral hepatitis, eGFR <60 mL/min/1.73 m², and albuminuria when available. GHF was defined by a strict “> cutoff” rule. The age/sex/BSA residual sensitivity used a weighted healthy-reference residual P95 cutoff of 15.24 mL/min/1.73 m² (reference-model n=2476). Legacy de-indexed thresholds are shown only for sensitivity analysis.

**Supplementary Table S4. Sensitivity of liver-phenotype associations to alternative GHF definitions and body-size handling.**

| **Study** | **Outcome definition** | **Contrast** | **N** | **Events** | **OR (95% CI)** | **P value** |
| --- | --- | --- | --- | --- | --- | --- |
| NHANES | Primary central Model 3 | G2 vs G1 | 7959 | 583 | 2.19 (1.57-3.07) | <0.001 |
| NHANES | Primary central Model 3 | G3 vs G1 | 7959 | 583 | 2.46 (1.51-4.00) | <0.001 |
| NHANES | Primary central Model 3 | G3 vs G2 | 7959 | 583 | 1.12 (0.74-1.70) | 0.596 |
| NHANES | Multiple imputation, Model 3 | G2 vs G1 | 9140 | 698 | 2.20 (1.62-2.99) | <0.001 |
| NHANES | Multiple imputation, Model 3 | G3 vs G1 | 9140 | 698 | 2.52 (1.61-3.93) | <0.001 |
| NHANES | Multiple imputation, Model 3 | G3 vs G2 | 9140 | 698 | 1.14 (0.78-1.68) | 0.499 |
| NHANES | Exclude active viral hepatitis | G2 vs G1 | 7884 | 576 | 2.14 (1.48-3.09) | <0.001 |
| NHANES | Exclude active viral hepatitis | G3 vs G1 | 7884 | 576 | 2.50 (1.56-4.02) | <0.001 |
| NHANES | Exclude active viral hepatitis | G3 vs G2 | 7884 | 576 | 1.17 (0.78-1.74) | 0.444 |
| NHANES | Indexed eGFR > age/sex P90 | G2 vs G1 | 7959 | 929 | 1.87 (1.47-2.38) | <0.001 |
| NHANES | Indexed eGFR > age/sex P90 | G3 vs G1 | 7959 | 929 | 2.17 (1.49-3.16) | <0.001 |
| NHANES | Indexed eGFR > age/sex P90 | G3 vs G2 | 7959 | 929 | 1.16 (0.78-1.73) | 0.463 |
| NHANES | Indexed-eGFR residual > P95 after age/sex/BSA model | G2 vs G1 | 7959 | 1083 | 2.14 (1.76-2.59) | <0.001 |
| NHANES | Indexed-eGFR residual > P95 after age/sex/BSA model | G3 vs G1 | 7959 | 1083 | 2.29 (1.71-3.07) | <0.001 |
| NHANES | Indexed-eGFR residual > P95 after age/sex/BSA model | G3 vs G2 | 7959 | 1083 | 1.07 (0.77-1.49) | 0.677 |
| NHANES | De-indexed eGFR > age/sex P95 | G2 vs G1 | 7959 | 1411 | 2.06 (1.70-2.50) | <0.001 |
| NHANES | De-indexed eGFR > age/sex P95 | G3 vs G1 | 7959 | 1411 | 2.10 (1.47-2.99) | <0.001 |
| NHANES | De-indexed eGFR > age/sex P95 | G3 vs G2 | 7959 | 1411 | 1.02 (0.72-1.45) | 0.919 |
| China | Primary: fixed NHANES indexed P95 | G2 vs G1 | 721 | 127 | 1.02 (0.60-1.71) | 0.950 |
| China | Primary: fixed NHANES indexed P95 | G3 vs G1 | 721 | 127 | 3.54 (1.39-9.02) | 0.008 |
| China | Primary: fixed NHANES indexed P95 | G3 vs G2 | 721 | 127 | 3.48 (1.31-9.22) | 0.012 |
| China | Fixed NHANES indexed P90 | G2 vs G1 | 721 | 176 | 0.88 (0.56-1.39) | 0.597 |
| China | Fixed NHANES indexed P90 | G3 vs G1 | 721 | 176 | 2.29 (0.95-5.50) | 0.065 |
| China | Fixed NHANES indexed P90 | G3 vs G2 | 721 | 176 | 2.59 (1.04-6.43) | 0.041 |
| China | Fixed NHANES de-indexed P95 | G2 vs G1 | 721 | 22 | 3.28 (1.17–9.15) | 0.024 |
| China | Fixed NHANES de-indexed P95 | G3 vs G1 | 721 | 22 | 2.11 (0.38–11.73) | 0.393 |
| China | Fixed NHANES de-indexed P95 | G3 vs G2 | 721 | 22 | 0.64 (0.13–3.12) | 0.585 |
| China | Fixed NHANES age/sex/BSA residual P95 | G2 vs G1 | 721 | 64 | 1.49 (0.81-2.74) | 0.199 |
| China | Fixed NHANES age/sex/BSA residual P95 | G3 vs G1 | 721 | 64 | 4.69 (1.61-13.71) | 0.005 |
| China | Fixed NHANES age/sex/BSA residual P95 | G3 vs G2 | 721 | 64 | 3.15 (1.08-9.21) | 0.036 |
| China | China-internal recalibrated age/sex residual P95 | G2 vs G1 | 721 | 45 | 0.91 (0.35-2.37) | 0.850 |
| China | China-internal recalibrated age/sex residual P95 | G3 vs G1 | 721 | 45 | 5.07 (1.60-16.00) | 0.006 |
| China | China-internal recalibrated age/sex residual P95 | G3 vs G2 | 721 | 45 | 5.55 (1.47-20.97) | 0.011 |

The indexed P95 outcome is primary. Indexed P90, an indexed-eGFR residual definition including age, sex, and BSA, and the legacy de-indexed P95 definition are sensitivity analyses. Fixed NHANES thresholds were transported to the Chinese cohort; the internally recalibrated Chinese residual P95 is a calibration-only analysis.

**Supplementary Table S5. Distribution of all observed liver-phenotype combinations before restriction to the primary G1/G2/G3 analysis.**

| **Phenotype combination** | **NHANES n** | **China n** |
| --- | --- | --- |
| MASLD / no significant fibrosis | 2703 | 178 |
| MASLD / significant fibrosis | 702 | 29 |
| No steatosis / no significant fibrosis | 5735 | 514 |
| No steatosis / significant fibrosis | 313 | 126 |
| Steatosis / CMRF status incomplete | 8 | 6 |

This table makes explicit the groups outside the primary three-level phenotype, particularly participants with significant fibrosis but no steatosis/MASLD phenotype and participants with incomplete cardiometabolic-risk-factor status. These participants are retained in separate all-participant sensitivity analyses rather than silently assigned to G1.

**Supplementary Table S6. VCTE quality-control metrics and probe distribution in NHANES.**

**Panel A. VCTE completion and reliability metrics**

| **NHANES cycle** | **Age-eligible examined, n** | **Complete VCTE, n (%)** | **≥10 valid measures, n** | **IQR/median <30%, n** | **Median IQR/median, %** | **Median success rate, %** |
| --- | --- | --- | --- | --- | --- | --- |
| 2017-March 2020 | 7881 | 6986 (88.6%) | 6986 | 6986 | 13.00 | 100 |
| August 2021-August 2023 | 5681 | 5020 (88.4%) | 5020 | 5020 | 13.65 | 100 |

**Panel B. Probe distribution**

| **NHANES cycle** | **M probe, n (%)** | **XL probe, n (%)** |
| --- | --- | --- |
| 2017-March 2020 | 5085 (72.8%) | 1900 (27.2%) |
| August 2021-August 2023 | 3788 (75.5%) | 1231 (24.5%) |

NHANES complete examinations had at least 10 valid measurements and IQR/median <30%; the corrected QC uses the NHANES IQR/median variable as a percentage. Probe type is reported because obesity differs by phenotype. The current result package does not contain equivalent acquisition-level QC fields for the Chinese clinical cohort; those procedural details should be added from the hospital VCTE protocol rather than inferred.

**Supplementary Table S7. Missing-data assessment, complete-case comparison, and multiple-imputation sensitivity.**

**Panel A. NHANES Model 3 covariate missingness**

| **NHANES Model 3 variable** | **N** | **Missing n** | **Percent missing** |
| --- | --- | --- | --- |
| Poverty-income ratio | 9140 | 1138 | 12.45% |
| Body mass index | 9140 | 34 | 0.37% |
| Marital status | 9140 | 8 | 0.09% |
| Current smoking | 9140 | 7 | 0.08% |
| Education level | 9140 | 6 | 0.07% |
| Race/ethnicity | 9140 | 0 | 0.00% |

**Panel B. NHANES complete-case versus excluded participants**

| **Group** | **N** | **GHF events** | **Unweighted GHF, %** | **Survey-weighted GHF, %** | **Mean age, years** |
| --- | --- | --- | --- | --- | --- |
| Model-3 complete case | 7959 | 583 | 7.33 | 6.44 | 50.05 |
| Excluded from complete-case Model 3 | 1181 | 115 | 9.74 | 9.24 | 50.64 |

**Panel C. Complete-case versus multiple-imputation Model 3 estimates**

| **Contrast** | **Complete-case OR (95% CI)** | **MI OR (95% CI)** | **MI P value** | **OR change, %** |
| --- | --- | --- | --- | --- |
| G2 vs G1 | 2.19 (1.57–3.07) | 2.20 (1.62–2.99) | ＜0.001 | 0.5 |
| G3 vs G1 | 2.46 (1.51–4.00) | 2.52 (1.61–3.93) | ＜0.001 | 2.5 |
| G3 vs G2 | 1.12 (0.74–1.70) | 1.14 (0.78–1.68) | 0.499 | 2.0 |

**Panel D. Missingness in the Chinese core study**

| **Chinese core variable** | **N** | **Missing n** | **Percent missing** |
| --- | --- | --- | --- |
| HbA1c | 853 | 672 | 78.78% |
| Triglycerides | 853 | 3 | 0.35% |
| HDL-C | 853 | 3 | 0.35% |
| Any cardiometabolic risk factor (CMRF) variable | 853 | 122 | 14.30% |

Fasting glucose and triglyceride absence in NHANES reflects the planned fasting subsample and is handled with fasting-subsample weights in Model 4 rather than ordinary item-missingness imputation. The main Model 3 complete-case analysis is accompanied by a 30-imputation survey-weighted sensitivity analysis.

**Supplementary Table S8. Medication use by liver phenotype and medication-related sensitivity analyses.**

**Panel A. NHANES 2017–March 2020 medication prevalence by liver phenotype**

| **Medication class** | **Phenotype** | **Users/N** | **Survey-weighted prevalence, %** |
| --- | --- | --- | --- |
| SGLT-2 inhibitor | G1 | 17/2973 | 0.4 (0.1–0.6) |
| SGLT-2 inhibitor | G2 | 23/1491 | 1.1 (0.5–1.7) |
| SGLT-2 inhibitor | G3 | 9/360 | 4.4 (0.4–8.3) |
| GLP-1 receptor agonist | G1 | 13/2973 | 0.2 (0.0–0.4) |
| GLP-1 receptor agonist | G2 | 16/1491 | 0.8 (0.2–1.3) |
| GLP-1 receptor agonist | G3 | 12/360 | 3.3 (0.4–6.2) |
| RAS inhibitor | G1 | 467/2973 | 12.0 (10.2–13.7) |
| RAS inhibitor | G2 | 433/1491 | 27.6 (23.6–31.6) |
| RAS inhibitor | G3 | 147/360 | 39.1 (31.1–47.1) |
| Any diuretic | G1 | 255/2973 | 6.5 (5.3–7.8) |
| Any diuretic | G2 | 253/1491 | 16.1 (13.9–18.3) |
| Any diuretic | G3 | 104/360 | 25.0 (16.9–33.2) |

**Panel B. Chinese clinical study medication prevalence**

| **Medication class** | **Overall** | **G1** | **G2** | **G3** | **P value** |
| --- | --- | --- | --- | --- | --- |
| SGLT2 inhibitor | 14 (1.9%) | 6 (1.2%) | 6 (3.4%) | 2 (6.9%) | 0.027 |
| GLP-1 receptor agonist | 0 (0.0%) | 0 (0.0%) | 0 (0.0%) | 0 (0.0%) | — |
| RAS inhibitor | 74 (10.3%) | 42 (8.2%) | 27 (15.2%) | 5 (17.2%) | 0.011 |
| Diuretic | 18 (2.5%) | 8 (1.6%) | 8 (4.5%) | 2 (6.9%) | 0.024 |

**Panel C. Medication exclusion/adjustment sensitivity analyses**

| **Study** | **Analysis** | **Contrast** | **N** | **Events** | **OR (95% CI)** | **P value** |
| --- | --- | --- | --- | --- | --- | --- |
| NHANES 2017–2020 | Exclude SGLT-2 inhibitor users | G2 vs G1 | 4775 | 381 | 2.42 (1.55–3.77) | ＜0.001 |
| NHANES 2017–2020 | Exclude SGLT-2 inhibitor users | G3 vs G1 | 4775 | 381 | 3.37 (1.85–6.14) | ＜0.001 |
| NHANES 2017–2020 | Exclude SGLT-2 inhibitor users | G3 vs G2 | 4775 | 381 | 1.39 (0.86–2.26) | 0.180 |
| NHANES 2017–2020 | Exclude GLP-1 receptor agonist users | G2 vs G1 | 4783 | 384 | 2.40 (1.51–3.80) | ＜0.001 |
| NHANES 2017–2020 | Exclude GLP-1 receptor agonist users | G3 vs G1 | 4783 | 384 | 3.09 (1.67–5.71) | ＜0.001 |
| NHANES 2017–2020 | Exclude GLP-1 receptor agonist users | G3 vs G2 | 4783 | 384 | 1.29 (0.76–2.19) | 0.349 |
| NHANES 2017–2020 | Exclude RAS inhibitor users | G2 vs G1 | 3777 | 306 | 2.47 (1.45–4.21) | ＜0.001 |
| NHANES 2017–2020 | Exclude RAS inhibitor users | G3 vs G1 | 3777 | 306 | 3.48 (1.54–7.87) | 0.003 |
| NHANES 2017–2020 | Exclude RAS inhibitor users | G3 vs G2 | 3777 | 306 | 1.41 (0.77–2.58) | 0.268 |
| NHANES 2017–2020 | Exclude diuretic users | G2 vs G1 | 4212 | 350 | 2.28 (1.36–3.84) | 0.002 |
| NHANES 2017–2020 | Exclude diuretic users | G3 vs G1 | 4212 | 350 | 3.79 (1.92–7.48) | ＜0.001 |
| NHANES 2017–2020 | Exclude diuretic users | G3 vs G2 | 4212 | 350 | 1.66 (1.05–2.64) | 0.032 |
| Chinese replication study | Exclude SGLT2i | G2 vs G1 | 707 | 125 | 1.01 (0.59–1.71) | 0.972 |
| Chinese replication study | Exclude SGLT2i | G3 vs G1 | 707 | 125 | 3.93 (1.52–10.20) | 0.005 |
| Chinese replication study | Exclude SGLT2i | G3 vs G2 | 707 | 125 | 3.89 (1.44–10.51) | 0.007 |
| Chinese replication study | Exclude SGLT2i or GLP-1RA | G2 vs G1 | 707 | 125 | 1.01 (0.59–1.71) | 0.972 |
| Chinese replication study | Exclude SGLT2i or GLP-1RA | G3 vs G1 | 707 | 125 | 3.93 (1.52–10.20) | 0.005 |
| Chinese replication study | Exclude SGLT2i or GLP-1RA | G3 vs G2 | 707 | 125 | 3.89 (1.44–10.51) | 0.007 |
| Chinese replication study | C3 + RAS inhibitor adjustment | G2 vs G1 | 721 | 127 | 1.03 (0.61–1.73) | 0.915 |
| Chinese replication study | C3 + RAS inhibitor adjustment | G3 vs G1 | 721 | 127 | 3.59 (1.40–9.18) | 0.008 |
| Chinese replication study | C3 + RAS inhibitor adjustment | G3 vs G2 | 721 | 127 | 3.49 (1.31–9.28) | 0.012 |

NHANES medication-class analyses are restricted to the 2017–March 2020 cycle because ascertainment is not fully harmonized across cycles. SGLT2 inhibitor exclusion is highlighted as the key medication sensitivity; RAS-inhibitor and diuretic analyses are exploratory.

**Supplementary Table S9. Significant fibrosis among participants with MASLD and CAP-conditioned analyses.**

**Panel A. Within-MASLD fibrosis estimates**

| **Study** | **Analysis/model** | **Exposure or test** | **N/events** | **OR (95% CI)** | **P value** |
| --- | --- | --- | --- | --- | --- |
| NHANES study | Event distribution | MASLD without significant fibrosis | 2703/293 | Reference | — |
| NHANES study | Event distribution | MASLD with significant fibrosis | 702/83 | — | — |
| NHANES study | Central Model 3 | Significant fibrosis vs no significant fibrosis | 2976/315 | 1.14 (0.73-1.79) | 0.558 |
| NHANES study | Central Model 3 + linear CAP | Significant fibrosis vs no significant fibrosis | 2976/315 | 1.11 (0.70-1.77) | 0.648 |
| NHANES study | Central Model 3 + RCS(CAP) | Significant fibrosis vs no significant fibrosis | 2976/315 | 1.13 (0.71-1.79) | 0.608 |
| NHANES study | Central Model 3 + RCS(CAP) | CAP overall association within MASLD, conditioned fibrosis model | — | — | 0.128 |
| NHANES study | Central Model 3 + RCS(CAP) | CAP nonlinearity within MASLD, conditioned fibrosis model | — | — | 0.247 |
| Chinese clinical study | Event distribution | MASLD without significant fibrosis | 178/26 | Reference | — |
| Chinese clinical study | Event distribution | MASLD with significant fibrosis | 29/8 | — | — |
| Chinese clinical study | Central C3 | Significant fibrosis vs no significant fibrosis | 207/34 | 3.94 (1.40-11.14) | 0.010 |
| Chinese clinical study | Central C3 + linear CAP | Significant fibrosis vs no significant fibrosis | 207/34 | 3.96 (1.40-11.21) | 0.009 |
| Chinese clinical study | Central C3 + spline CAP | Significant fibrosis vs no significant fibrosis | 207/34 | 4.61 (1.57-13.54) | 0.005 |

**Panel B. Collinearity diagnostics for the CAP-conditioned NHANES model**

| **Term** | **GVIF** | **Df** | **GVIF^(1/(2*Df))** |
| --- | --- | --- | --- |
| Significant fibrosis | 1.150 | 1 | 1.072 |
| CAP | 1.208 | 1 | 1.099 |
| BMI | 1.490 | 1 | 1.221 |

CAP-conditioned models test whether the significant-fibrosis contrast persists after accounting for steatosis severity. The weighted Pearson correlation between CAP and the significant-fibrosis indicator was 0.202; adjusted GVIF diagnostics were close to 1, arguing against problematic collinearity. These analyses are secondary and should not be described as demonstrating fibrosis-specific independence.

**Supplementary Table S10. Restricted cubic spline specification, support ranges, and tests.**

**Panel A. Knot locations, reference values, and supported exposure ranges**

| **Study** | **Exposure** | **Knots, n** | **Knot locations** | **Reference** | **Displayed support range** | **Unit** |
| --- | --- | --- | --- | --- | --- | --- |
| NHANES | CAP | 4 | 166.0, 234.0, 287.0, 375.0 | 260.0 | 166.0–375.0 | dB/m |
| NHANES | LSM | 4 | 3.1, 4.4, 5.5, 10.1 | 4.9 | 3.1–10.1 | kPa |
| Chinese replication cohort | CAP | 4 | 145.0, 221.0, 266.0, 337.4 | 242.0 | 145.0–337.4 | dB/m |
| Chinese replication cohort | LSM | 4 | 3.0, 4.1, 5.5, 21.1 | 4.7 | 3.0–21.1 | kPa |

**Panel B. Overall and nonlinearity tests**

| **Study** | **Exposure** | **Test** | **P value** | **N** | **Events** |
| --- | --- | --- | --- | --- | --- |
| NHANES | CAP | CAP RCS overall association | 3.27e-04 | 8236 | 604 |
| NHANES | CAP | CAP RCS nonlinearity | 0.124 | 8236 | 604 |
| NHANES | LSM | LSM RCS overall association | 0.677 | 8236 | 604 |
| NHANES | LSM | LSM RCS nonlinearity | 0.531 | 8236 | 604 |
| Chinese replication cohort | CAP | CAP overall | 0.677 | 853 | 160 |
| Chinese replication cohort | CAP | CAP nonlinearity | 0.562 | 853 | 160 |
| Chinese replication cohort | LSM | LSM overall | 0.023 | 853 | 160 |
| Chinese replication cohort | LSM | LSM nonlinearity | 0.046 | 853 | 160 |
| Chinese replication cohort | CAP | CAP conditional on LSM | 0.686 | 853 | 160 |
| Chinese replication cohort | LSM | LSM conditional on CAP | 0.023 | 853 | 160 |

Spline figures are displayed only over prespecified supported ranges and are accompanied by exposure distributions (Supplementary Figure S1A–S1D). NHANES used weighted knot locations; the Chinese fixed analyses use the locked external-replication dataset.

**Supplementary Table S11. FIB-4 sensitivity analyses in NHANES.**

| **Analysis** | **Contrast** | **N** | **Events** | **OR (95% CI)** | **P value** |
| --- | --- | --- | --- | --- | --- |
| FIB-4 alternative 3-group | F-G2 vs F-G1 | 8063 | 589 | 2.18 (1.57–3.02) | 3.20e-06 |
| FIB-4 alternative 3-group | F-G3 vs F-G1 | 8063 | 589 | 2.41 (0.74–7.80) | 0.143 |
| FIB-4 alternative 3-group | F-G3 vs F-G2 | 8063 | 589 | 1.11 (0.40–3.07) | 0.847 |
| Within-MASLD FIB-4 category | Intermediate vs Low | 2960 | 312 | 1.53 (1.05–2.23) | 0.028 |
| Within-MASLD FIB-4 category | High vs Low | 2960 | 312 | 1.51 (0.54–4.21) | 0.428 |
| Within-MASLD FIB-4 category | High vs Intermediate | 2960 | 312 | 0.99 (0.31–3.12) | 0.986 |
| Continuous log(FIB-4) | Per 1 weighted SD higher log(FIB-4) | 8191 | 597 | 0.75 (0.60–0.93) | 0.010 |
| Continuous log(FIB-4) | Per 1 weighted SD higher log(FIB-4) | 2960 | 312 | 0.84 (0.63–1.13) | 0.254 |

FIB-4 is used as an alternative non-invasive fibrosis marker to assess potential misclassification from VCTE-defined LSM ≥8 kPa. These analyses are supportive rather than replacements for VCTE.

**Supplementary Table S12. Exploratory blood-pressure analyses: subgroup counts and interaction tests.**

**Panel A. Participant and GHF-event counts in every liver-phenotype × blood-pressure cell**

| **Study** | **Liver phenotype** | **BP category** | **N** | **GHF events** | **Non-events** |
| --- | --- | --- | --- | --- | --- |
| NHANES | G1 | Normal | 2527 | 134 | 2393 |
| NHANES | G1 | Elevated | 603 | 38 | 565 |
| NHANES | G1 | Hypertension | 2368 | 123 | 2245 |
| NHANES | G2 | Normal | 698 | 81 | 617 |
| NHANES | G2 | Elevated | 243 | 33 | 210 |
| NHANES | G2 | Hypertension | 1648 | 162 | 1486 |
| NHANES | G3 | Normal | 111 | 12 | 99 |
| NHANES | G3 | Elevated | 40 | 8 | 32 |
| NHANES | G3 | Hypertension | 531 | 61 | 470 |
| Chinese replication study | G1 | Normal | 294 | 60 | 234 |
| Chinese replication study | G1 | Elevated | 94 | 14 | 80 |
| Chinese replication study | G1 | Hypertension | 126 | 19 | 107 |
| Chinese replication study | G2 | Normal | 71 | 12 | 59 |
| Chinese replication study | G2 | Elevated | 33 | 5 | 28 |
| Chinese replication study | G2 | Hypertension | 74 | 9 | 65 |
| Chinese replication study | G3 | Normal | 6 | 2 | 4 |
| Chinese replication study | G3 | Elevated | 10 | 5 | 5 |
| Chinese replication study | G3 | Hypertension | 13 | 1 | 12 |

**Panel B. Interaction tests**

| **Study** | **Interaction analysis** | **N** | **Events** | **P for interaction** | **Interpretation** |
| --- | --- | --- | --- | --- | --- |
| NHANES | 3-level liver phenotype × 3-level BP | 7675 | 554 | 0.477 | Survey-weighted Wald interaction |
| Chinese replication study | 3-level liver phenotype × 3-level BP | 721 | 127 | Not reliably estimable | Sparse G3 × BP cells |
| Chinese replication study | Firth penalized 3-level liver phenotype × 3-level BP | 721 | 127 | 0.160 | Additional sparse-data sensitivity analysis |
| Chinese replication study | MASLD yes/no × 3-level BP | 847 | 159 | 0.314 | Exploratory |

Blood-pressure interaction analyses are exploratory. The primary NHANES three-level interaction was not significant. In the Chinese cohort, the conventional three-level interaction was not considered reliably estimable because G3 cells were sparse; a subsequently performed Firth penalized sensitivity analysis yielded P for interaction=0.160. Differences between stratum-specific ORs should not be interpreted as effect modification when the interaction test is non-significant.

**Supplementary Table S13. Harmonized cross-study and four-phenotype analyses.**

**Panel A. Harmonized common-covariate models**

| **Study** | **Contrast** | **N** | **Events** | **OR (95% CI)** | **P value** |
| --- | --- | --- | --- | --- | --- |
| NHANES preferred (+ survey cycle) | G2 vs G1 | 9099 | 692 | 2.64 (1.96–3.55) | ＜0.001 |
| NHANES strict common | G2 vs G1 | 9099 | 692 | 2.65 (1.97–3.56) | ＜0.001 |
| China common model | G2 vs G1 | 721 | 127 | 1.02 (0.60–1.71) | 0.950 |
| NHANES preferred (+ survey cycle) | G3 vs G1 | 9099 | 692 | 3.16 (2.06–4.84) | ＜0.001 |
| NHANES strict common | G3 vs G1 | 9099 | 692 | 3.15 (2.05–4.85) | ＜0.001 |
| China common model | G3 vs G1 | 721 | 127 | 3.54 (1.39–9.02) | 0.008 |
| NHANES preferred (+ survey cycle) | G3 vs G2 | 9099 | 692 | 1.20 (0.84–1.71) | 0.323 |
| NHANES strict common | G3 vs G2 | 9099 | 692 | 1.19 (0.84–1.70) | 0.327 |
| China common model | G3 vs G2 | 721 | 127 | 3.48 (1.31–9.22) | 0.012 |

**Panel B. Cross-study heterogeneity**

| **Comparison / contrast** | **P for heterogeneity** |
| --- | --- |
| Preferred: G2 vs G1 | 0.002 |
| Preferred: G3 vs G1 | 0.828 |
| Preferred: G3 vs G2 | 0.044 |
| Strict common: G2 vs G1 | 0.002 |
| Strict common: G3 vs G1 | 0.828 |
| Strict common: G3 vs G2 | 0.043 |

**Panel C. Four-phenotype analysis**

| **Study** | **Contrast** | **N** | **Events** | **OR (95% CI)** | **P value** |
| --- | --- | --- | --- | --- | --- |
| NHANES | P2 vs P1: steatosis/no fibrosis vs neither | 9416 | 719 | 2.65 (1.97–3.56) | ＜0.001 |
| NHANES | P3 vs P1: fibrosis/no steatosis vs neither | 9416 | 719 | 2.57 (1.34–4.93) | 0.005 |
| NHANES | P4 vs P1: steatosis+fibrosis vs neither | 9416 | 719 | 3.19 (2.09–4.86) | ＜0.001 |
| NHANES | P4 vs P2: fibrosis contrast among steatosis | 9416 | 719 | 1.20 (0.85–1.71) | 0.301 |
| NHANES | P4 vs P3: steatosis contrast among fibrosis | 9416 | 719 | 1.24 (0.62–2.50) | 0.545 |
| NHANES | P3 vs P2: fibrosis-only vs steatosis-only | 9416 | 719 | 0.97 (0.53–1.79) | 0.923 |
| China | P2 vs P1: steatosis/no fibrosis vs neither | 853 | 160 | 0.95 (0.58–1.57) | 0.846 |
| China | P3 vs P1: fibrosis/no steatosis vs neither | 853 | 160 | 1.93 (1.18–3.14) | 0.008 |
| China | P4 vs P1: steatosis+fibrosis vs neither | 853 | 160 | 3.26 (1.31–8.12) | 0.011 |
| China | P4 vs P2: fibrosis contrast among steatosis | 853 | 160 | 3.43 (1.32–8.94) | 0.012 |
| China | P4 vs P3: steatosis contrast among fibrosis | 853 | 160 | 1.70 (0.64–4.46) | 0.285 |
| China | P3 vs P2: fibrosis-only vs steatosis-only | 853 | 160 | 2.02 (1.09–3.77) | 0.026 |

These analyses are moved from the main manuscript to the supplement because the Chinese G3 subgroup is small and the reviewer requested more cautious interpretation of cross-study replication. Preferred harmonized models use age, sex, current smoking, and BMI in both cohorts, with NHANES additionally adjusted for survey cycle; a strict covariate-identical NHANES sensitivity model omits survey cycle.

**Supplementary Table S14. Standardized definitions of the primary outcome, liver phenotypes, alcohol eligibility, and adjustment models.**

| **Item** | **Definition** |
| --- | --- |
| Primary GHF | Indexed eGFR above the survey-weighted age- and sex-specific 95th percentile derived in the pooled NHANES low-risk reference (10-year age bands). The same fixed cutoffs were applied to the Chinese study. |
| Model 1 / C1 | Age and sex. |
| Model 2 | NHANES: Model 1 + race/ethnicity, poverty-income ratio, education, current smoking, and marital status. |
| C2 | Chinese study: C1 + current smoking. |
| Model 3 / C3 | Corresponding Model 2/C2 + BMI. This is the central body-size-adjusted model. |
| Model 4 / C4 | Corresponding Model 3/C3 + fasting plasma glucose, log-transformed triglycerides, hypertension, and HDL-C; NHANES uses fasting-subsample weights and same-sample comparisons. |
| Harmonized model | Age, sex, current smoking, and BMI in both cohorts; NHANES additionally includes survey cycle and the complex survey design. |
| Liver phenotypes | G1: no steatosis/no significant fibrosis; G2: MASLD without significant fibrosis; G3: MASLD with significant fibrosis. |
| Alcohol eligibility | Women <140 g/week and men <210 g/week; participants with unknown alcohol exposure were excluded from the primary alcohol-corrected analytic study. |

This table is intended to prevent ambiguity across the manuscript, supplement, figures, and response letter. It also standardizes terminology to “de-indexed eGFR” rather than “absolute GFR.”

## 1.2 Supplementary Figures

**Supplementary Figure S1A. NHANES CAP restricted cubic spline with exposure distribution.**

The lower panel shows the survey-weighted CAP distribution. Knot locations, reference values, and the supported exposure range are reported in Supplementary Table S10.

**Supplementary Figure S1B. NHANES LSM restricted cubic spline with exposure distribution.**

The lower panel shows the survey-weighted LSM distribution. Estimates outside the prespecified supported range are not emphasized.

**Supplementary Figure S1C. Chinese clinical study CAP restricted cubic spline with exposure distribution.**

The lower panel shows the observed CAP distribution in the Chinese clinical study.

**Supplementary Figure S1D. Chinese clinical study LSM restricted cubic spline with exposure distribution.**

The lower panel shows the observed LSM distribution in the Chinese clinical study.

**Supplementary Figure S2. Exploratory blood-pressure-stratified association in NHANES.**

These subgroup estimates are exploratory. The global liver-phenotype × three-level blood-pressure interaction was not statistically significant (P=0.477).

**Supplementary Figure S3. Harmonized cross-study comparison.**

Harmonized models use common covariates to compare effect estimates across cohorts. This figure is supplementary because the Chinese G3 subgroup is small and fibrosis-specific estimates are imprecise.

**Supplementary Figure S4. Four-phenotype analysis of steatosis and significant fibrosis across study.**

This sensitivity analysis retains all steatosis/fibrosis combinations and directly addresses the possibility that a three-level MASLD phenotype omits clinically relevant groups.
